# JunB promotes Th17 cell identity and restrains alternative CD4^+^ T-cell programs during inflammation

**DOI:** 10.1038/s41467-017-00380-3

**Published:** 2017-08-21

**Authors:** Tiffany M. Carr, Joshua D. Wheaton, Geoffrey M. Houtz, Maria Ciofani

**Affiliations:** Department of Immunology, Duke University Medical Center, Durham, North Carolina 27710 USA

## Abstract

T helper 17 (Th17) cell plasticity contributes to both immunity and autoimmunity; however, the factors that control lineage flexibility are mostly unknown. Here we show the activator protein-1 (AP-1) factor JunB is an essential regulator of Th17 cell identity. JunB activates expression of Th17 lineage-specifying genes and coordinately represses genes controlling Th1 and regulatory T-cell fate. JunB supports Th17 cell identity by regulating key AP-1 complex constituents. In particular, JunB limits the expression of the subset repressor IRF8, and impedes access of JunD to regulatory regions of alternative effector loci. Although dispensable for homeostatic Th17 cell development, JunB is required for induction and maintenance of Th17 effector responses in the inflammatory contexts of both acute infection and chronic autoimmunity in mice. Through regulatory network analysis, we show that JunB is a core regulator of global transcriptional programs that promote Th17 cell identity and restrict alternative CD4^+^ T-cell potential.

## Abstract (web summary)

AP-1 family transcription factors regulate CD4^+^ T helper cell differentiation. Here the authors show that the AP-1 member JunB is a nonredundant regulator of transcriptional programs that support Th17 cell identity and restrain alternative Th1 and Treg cell fates in inflammatory contexts of acute fungal infection and chronic autoimmunity.

## Introduction

Functional plasticity in immune cells enhances the adaptability of responses targeting pathogens, but can also be detrimental to the host. Upon antigenic stimulation, CD4^+^ T cells adopt one of two opposing fates: a helper T (Th) cell specialized in supporting the clearance of infections, or a regulatory T (Treg) cell that functions to attenuate immune responses. Cytokines and other microenvironmental ligands present during T-cell activation direct diverse effector Th and Treg cell differentiation programs via the induction of function-specifying transcription factors (TF). The resulting subsets include Treg cells defined by Foxp3 expression, Th1 cells defined by T-bet (*Tbx21*) and IFNγ production, Th2 cells defined by GATA3 and IL-4 secretion, and Th17 cells defined by RORγt and IL-17 production. The identification of CD4^+^ T cells that co-express TFs and cytokines associated with alternative subsets highlights the vast flexibility inherent to CD4^+^ T-cell diversification^1^.

Among this diversity, Th17 cells stand out as particularly flexible in acquiring features of multiple other CD4^+^ T-cell subclasses under altered cytokine conditions^2, 3^. At steady state, Th17 cells secreting IL-17 and IL-22 occupy mucosal barriers such as the intestinal lamina propria, where they regulate tissue homeostasis by promoting epithelial barrier function^4^. During infection, Th17 cells provide critical support for immunity against extracellular bacteria and fungi, and can readily adopt Th1-like characteristics (e.g., T-bet and IFNγ expression) in such inflammatory settings^5, 6^. Although advantageous during infection, Th17 cell effector flexibility is also associated with deleterious Th17 cell function in autoimmunity. Indeed, pathogenic IFNγ^+^ IL-17A^+^ cells have been identified in numerous disease settings, including multiple sclerosis (MS) and inflammatory bowel disease (IBD)^7, 8^, and exacerbate inflammatory disease in mouse models of these autoimmune conditions^9–11^. Moreover, genetic fate mapping tools in mice have provided convincing evidence for the context-dependent in vivo conversion of Th17 cells into T follicular helper cells in intestinal Peyer’s patches^12^, IFNγ^+^ Th1-like cells in an autoimmune model of MS^11^, and T regulatory type 1 cells (Tr1) during resolution of inflammation^13^. In light of the pervasive role of plasticity in CD4^+^ T-cell function and dysfunction, an understanding of the underlying mechanisms is of critical importance.

Th17 cell identity is governed by a network of transcriptional regulators that provide dynamic programming downstream of environmental inputs^14, 15^. While the central regulator RORγt is essential for the expression of a limited set of key subset effectors^14, 16^, additional TFs are required to specify the full Th17 cell program. In particular, a core of obligatory initiator TFs including STAT3^17^, IRF4^18^, and BATF^19^ integrate cytokine (IL-6/IL-21/IL-23) and T-cell receptor (TCR) signals to coordinately activate Th17 cell differentiation^14^. Beyond this, less is appreciated about the molecular mechanisms that sustain subset identity and that facilitate functional plasticity. Early studies suggested the flexibility of Th17 cells is facilitated by a permissive, bivalent histone epigenetic status at polarizing TF loci—such as *Tbx21* and *Gata3—*that may allow for subset reversibility and reprogramming^20^. Although epigenetic modulators that control effector status in Th17 cells have been identified^15, 21^, the TF regulators that globally program the capacity of CD4^+^ T cells to dynamically control their functional identity in response to changing contexts are mostly undefined.

TFs of the activation protein-1 (AP-1) family are compelling candidates for mediating CD4^+^ T-cell identity switches. AP-1 factors are basic leucine-zipper TFs comprised of homodimers or heterodimers of Jun with Fos, or ATF protein classes. These TFs are dynamically regulated by extracellular signals and can either activate or repress transcription. AP-1 consensus motifs predominate in global analyses of Th17 cell *cis* regulatory regions^14, 22^, which coincides with important functional roles for AP-1 TFs in Th17 cell differentiation. In particular, BATF and its cooperative binding partner IRF4^14, 23, 24^, are essential pioneer factors that establish chromatin accessibility at Th17 regulatory regions downstream of TCR signals. This activity pre-patterns the enhancer landscape for further subset-selective gene regulation^14^. Accordingly, BATF collaborates in high-order regulatory complexes with other Th17 specifying TFs, including STAT3, linking TCR and cytokine signals to epigenetic changes^14, 22^. The identification of Fosl2 as a broad repressor of Th effector genes in Th17 cells adds another layer of complexity in the Th17 AP-1 network^14^. Fosl2 restricts Th1 and Treg cell potential, yet also antagonizes important Th17 program genes (e.g. *Il17a/f*, *Ccl20*, *Ccr6*)^14^, suggesting balancing contributions by other AP-1 factors in the regulation of Th17 cell plasticity. In this regard, JunB is a dominant AP-1 dimerization partner for both BATF and Fosl2 in Th17 cells^19, 24^, with an established role in CD4^+^ T-cell differentiation. In Th2 cells, JunB is critical for *Il4* transcription, while also restricting inappropriate *Ifng* expression^25^, intimating a function for JunB in physiological Th17 cell effector conversions. Indeed, JunB may be poised to sense shifts in environmental context as its protein levels are subject to dynamic control via posttranslational modification in CD4^+^ T cells^26, 27^. The contribution of JunB to Th17 cell differentiation and its regulation of effector identity within the growing Th17 cell TF network has not been evaluated.

Here, we identify JunB as a critical regulator of Th17 cell identity. Deletion of *Junb* in Th17 cell differentiation results in a marked reduction in IL-17A-producing cells and an aberrant emergence of Th1-like and iTreg-like cells. Although dispensable for homeostatic Th17 cells, JunB is essential for induction of these cells in inflammatory settings. Specifically, in the absence of JunB, in vivo inflammation induced by infection with *Candida albicans* or a model antigen in the context of experimental autoimmune encephalomyelitis (EAE) results in impaired Th17 cell responses with an upregulation of a Th1 cell phenotype. Global analysis of JunB-dependent gene expression and genomic occupancy reveals that JunB controls Th17 cell stability through direct activation of important Th17 effector genes in concert with direct repression of subset-defining regulators of the Th1 and Treg cell lineages (e.g. *Tbx21*, *Foxp3*, and *Ifng*). Beyond these select genes, JunB functions as a core regulatory node in global programs that promote Th17 cell differentiation while constraining alternative effector and regulatory CD4^+^ T-cell potentials.

## Results

### JunB regulates Th17 cell identity

**Fig. 1:**
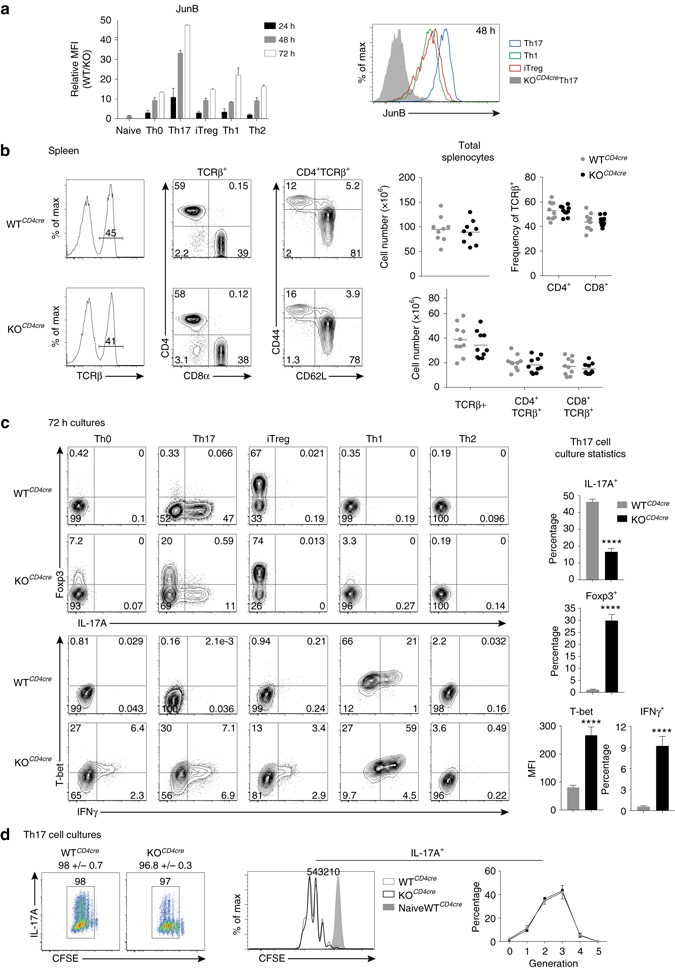
JunB promotes Th17 cell identity and represses Th1 and iTreg cell programs. **a** Flow cytometry of JunB expression in sort-purified *Junb* ^*+/+*^CD4cre (WT^*CD4cre*^) naive CD4^+^ T cells (CD4^+^CD25^−^CD44^lo^CD62L^+^) or WT^*CD4cre*^ naive CD4^+^ T cells cultured under Th0, Th17, iTreg, Th1, or Th2 conditions, for the indicated times. *Junb* ^*fl/fl*^CD4cre (KO^*CD4cre*^) naive CD4^+^ T cells were cultured and analyzed in concert as a negative control for staining. *Error bars* represent SEM of two independent experiments. Histograms depict levels of JunB in Th17 (*blue*), iTreg (*red*), and Th1 (*green*) cells, 48 h post polarization. **b** Flow cytometry of splenocytes from WT^*CD4cre*^ and KO^*CD4cre*^ mice, stained for TCRβ, CD4, and CD8α. Total splenocyte counts, frequencies of CD4^+^TCRβ^+^ and CD8^+^TCRβ^+^ splenocytes, and total numbers of TCRβ^+^ cells are shown. **c** Flow cytometry of WT^*CD4cre*^ and KO^*CD4cre*^ sort-purified naive CD4^+^ T cells cultured under Th0, Th17, iTreg, Th1, or Th2 conditions for 72 h followed by restimulation. Data are representative of ten independent experiments which are compiled into bar graphs. *Error bars* depict SEM. **d** Flow cytometric analysis of CFSE dilution in restimulated 72 h Th17 cell cultures relative to IL-17A expression (*left*), and specifically in IL-17A-expressing cells (*right*). The frequency of cells in each generation is indicated in the line graph. *Error bars* represent SEM of two independent experiments with at least five mice per group. ****p<0.0001 (unpaired two-tailed Student’s *t*-test)

To determine the expression profile of JunB in CD4^+^ T-cell subsets, we evaluated in vitro differentiated CD4^+^ T cells at 24, 48, and 72 h for levels of JunB. At each time point, the relative mean fluorescence intensity of JunB was on average 3-fold higher when naive CD4^+^ T cells were cultured under Th17 cell polarizing conditions (IL-6 and TGF-β), as compared to induced Treg (iTreg,TGFβ and IL-2), Th1 (IL-12), or Th2 (IL-4) cell polarizing conditions, or to non-polarizing Th0 conditions (TCR stimulation alone) (Fig. 1a). After 5 days, JunB remained most highly expressed in Th17 cells (Supplementary Fig. 1a). However, *Junb* transcript levels were not as differential, consistent with posttranslational mechanisms that regulate JunB protein turnover in CD4^+^ T cells^26, 27^ (Supplementary Fig. 1b). The selective early induction and sustained elevated levels of JunB in Th17 cells suggested that JunB plays an important role during Th17 cell differentiation.

To test this hypothesis, we bred mice with a conditional *Junb* allele^28^ to the CD4-Cre pan T-cell deleter strain^29^. First, we compared αβ T-cell subsets in naive *Junb* ^*+/+*^CD4-Cre (WT^*CD4cre*^) and *Junb* ^*fl/fl*^CD4-Cre (KO^*CD4cre*^) mice. Total thymocyte numbers and selection, based on TCRβ and CD69 expression, were unaffected in KO^*CD4cre*^ mice, and mature CD4^+^TCRβ^+^ and CD8α^+^TCRβ^+^ thymocytes developed in the expected frequencies (Supplementary Fig. 1c). In the spleen and mesenteric lymph nodes (LN), total cellularity, as well as the frequencies and numbers of CD4^+^TCRβ^+^ and CD8α^+^TCRβ^+^ cells were similar (Fig. 1b and Supplementary Fig. 1d). Importantly, the frequency of naive CD4^+^TCRβ^+^CD44^lo^CD62L^+^ cells was also unaltered in the periphery of KO^*CD4cre*^ mice (Fig. 1b and Supplementary Fig. 1d), permitting further study of effector diversification.

Th17 cells can be differentiated in vitro to mimic distinct functional classes. Exposure to IL-23 elicits pathogenic potential, such that Th17 cells polarized with IL-6, IL-1β, and IL-23 effectively induce disease compared to non-pathogenic Th17 cells polarized with IL-6 and TGFβ^30, 31^. Differentiation of naive CD4^+^ T cells purified from WT^*CD4cre*^ and KO^*CD4cre*^ mice with IL-6 and TGFβ revealed a striking dysregulation in cytokine and TF expression for mutant cells. JunB*-*deficient cultures displayed a 2-fold to 4-fold decrease in the proportion of IL-17A-expressing cells vs. WT^*CD4cre*^ cultures, as well as robust populations of aberrant Foxp3^+^ iTreg-like and T-bet^+^IFNγ^+^ Th1-like cells (Fig. 1c). IFNγ and IL-17A expression remained nearly mutally exclusive (Supplementary Fig. 1f). Importantly, the reduction in IL-17A^+^ cells is not due to a defect in culture proliferation, or a selective impairment of IL-17A^+^ cell proliferation or survival in the absence of JunB (Fig. 1d and Supplementary Fig. 1e). A similar dysregulation occurred using ‘pathogenic’ Th17 cell-promoting conditions (Supplementary Fig. 1g), although, KO^*CD4cre*^ cultures generated fewer Foxp3^+^ cells, suggesting that efficient release of iTreg-like potential requires TGFβ-mediated signals. Accordingly, we also observed less atypical Foxp3 expression in KO^*CD4cre*^ naive CD4^+^ T cells cultured without exogenous TGFβ in Th0 and Th1 conditions relative to ‘non-pathogenic’ KO^*CD4cre*^ Th17 conditions (Fig. 1c). In addition, the upregulated Th1-like T-bet^+^ IFNγ^+^ profile of KO^*CD4cre*^ Th17 cells also occurred in JunB-deficient Th0, iTreg, and Th1 cultures, suggesting a general role for JunB in limiting Th1 cell differentiation (Fig. 1c). Indeed, similar to studies using *Junb*-hypomorphic mice^25^, KO^*CD4cre*^ Th2 cell cultures produced reduced levels of IL-4 and increased IFNγ, compared to WT^*CD4cre*^ by day 5 (Supplementary Fig. 1h). Taken together, JunB has a nonredundant role in promoting Th17 cell identity and restraining alternative Th1 and Treg potential during Th17 cell induction.

### Th17 cells require continuous expression of JunB

**Fig. 2:**
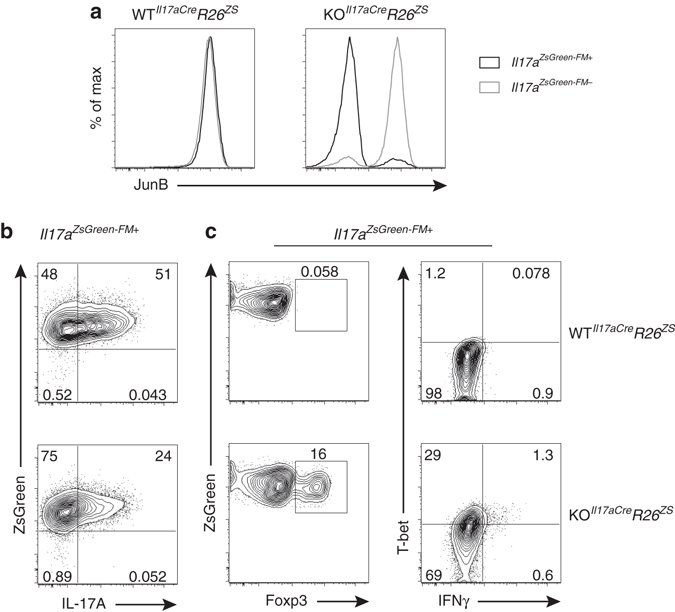
Continuous expression of JunB is required to maintain the Th17 cell phenotype. **a** Flow cytometry of JunB expression in *Il17a* ZsGreen^+^ fate-mapped (*Il17a* ^ZsGreen-FM+^) cells and *Il17a* ^ZsGreen-FM-^ cells from *Junb* ^*+/+*^ *Il17a* ^*cre+/*−^ *Rosa26* ^*ZSGreen+/*−^ (WT^*Il17aCre*^ *R26* ^*ZS*^) and *Junb* ^*fl/fl*^ *Il17a* ^*cre+/*−^ *Rosa26* ^*ZSGreen+/*−^ (KO^*Il17aCre*^ *R26* ^*ZS*^) Th17 cell cultures, day 4 post-polarization of naive CD4^+^ T cells. **b** Flow cytometry of *Il17a* ^ZsGreen-FM+^ cells sort purified from day 4 WT^*Il17aCre*^ *R26* ^*ZS*^ and KO^*Il17aCre*^ *R26* ^*ZS*^ Th17 cell cultures re-seeded in Th17 cell polarizing conditions for 4 days followed by restimulation. **c** Flow cytometry of intracellular IL-17A in *Il17a* ^ZsGreen-FM+^ cells, plated as in **b**, following stimulation with PMA and ionomycin. Data are representative of three independent experiments

To interrogate the role of JunB in the maintenance of Th17 cell effector identity we deleted *Junb* in differentiated Th17 cells and simultaneously employed a fate mapping strategy to track Th17 to Th1 or Treg cell conversion events. Specifically, conditional *Junb* mutant mice were bred with mice expressing *Cre* from the *Il17a* locus (*Il17a* ^*Cre/+*^)^11^, as well as with a reporter strain encoding a floxed-stop *Zsgreen* conditional allele in the ubiquitous *Rosa26* locus^32^. Consequently, ZsGreen expression permanently marked cells with both a history of *Il17a* expression and *Junb* gene deletion in *Junb* ^*fl/fl*^ cells (Fig. 2a).

Th17 cell plasticity under defined, altered conditions can be modeled in vitro^2, 3^. We differentiated naive CD4^+^ T cells from *Junb* ^*+/+*^ *Il17a* ^*Cre/+*^ *Rosa26* ^*ZsGreen/+*^ (WT^*Il17aCre*^ *R26* ^*ZS*^) and *Junb* ^*fl/fl*^ *Il17a* ^*Cre/+*^ *Rosa26* ^*ZsGreen/+*^ (KO^*Il17aCre*^ *R26* ^*ZS*^) mice under Th17 conditions for 4 days to generate *Il17a* ZsGreen^+^ fate-mapped cells (*Il17a* ^ZsGreen-FM+^). Purified *Il17a* ^ZsGreen-FM+^ cells were transferred back to Th17 conditions to evaluate the role of JunB in regulating Th17 cell stability, or alternatively, to altered cytokine conditions to establish maximal Th1 and iTreg lineage conversion potential. Compared to wild type, JunB-deficient *Il17a* ^ZsGreen-FM+^ cells preserved in Th17-promoting conditions, displayed a marked reduction in IL-17A and IL-17F expression (Fig. 2b and Supplementary Fig. 2c). Moreover, a prominent fraction of JunB-deficient *Il17a* ^ZsGreen-FM+^ cells underwent effector conversion to Treg- or Th1-like phenotypes (Fig. 2c). The extent of Treg re-direction was similar when *Il17a* ^ZsGreen-FM+^ cells were transferred to a potent iTreg-polarizing condition (Supplementary Fig. 2a), indicating that efficient transdifferentiation occurs when JunB expression is extinguished in Th17 cells. Conversely, the level of T-bet in JunB-deficient *Il17a* ^ZsGreen-FM+^ Th17 cells was higher for cells transferred to Th1 vs. Th17 cell conditions (Supplementary Fig. 2b), suggesting the full acquisition of Th1 status upon conversion is facilitated by altered cytokine inputs. Therefore, continuous JunB expression is required to both maintain Th17 cell effector identity and constrain plasticity towards alternative CD4^+^ T-cell fates in cells that have initated the Th17 cell program.

### JunB actively promotes Th17 cell identity

**Fig. 3:**
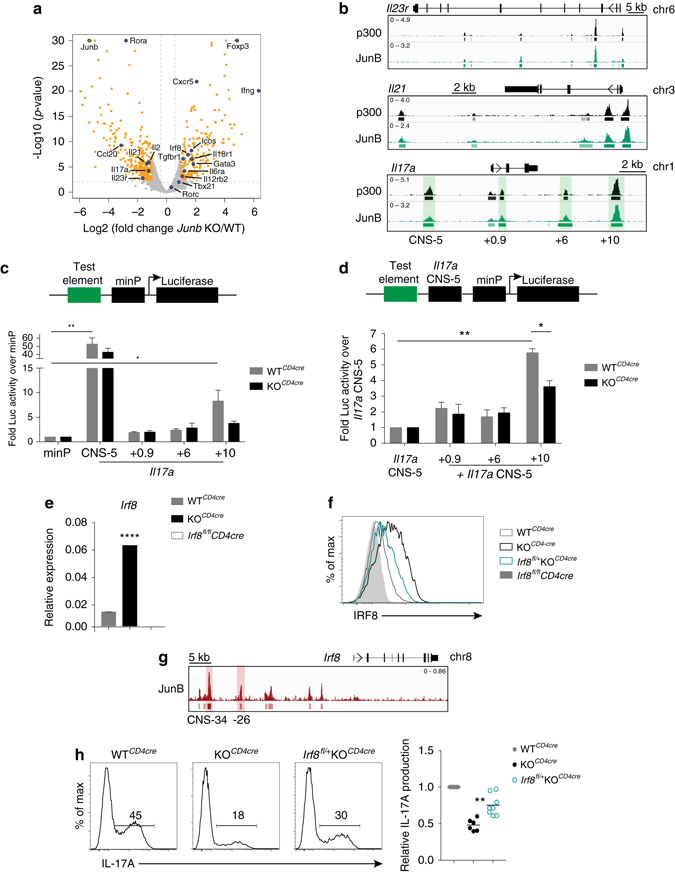
JunB actively promotes the Th17 cell program. **a** Differential mRNA expression in WT^*CD4cre*^ and KO^*CD4cre*^ 48 h Th17 cell polarization cultures, displayed as a volcano plot of log2 fold change vs. the –log10(p-value) for each gene. Genes considered significant below FDR < 0.05 are highlighted in *orange*, and select genes involved in T-cell differentiation are labeled and highlighted in *blue*. *Gray* trendlines indicate fold change of 1.5 and p-value = 0.01. p-value capped at 10^−30^. **b** JunB occupancy in 48 h polarized Th17 cells. ChIP-Seq tracks at the *Il23r*, *Il21*, and *Il17a* loci are displayed using the Integrative Genomics Viewer (IGV; Broad Institute). Lower track marks the boundaries of significant peaks called by MACS2 with FDR<0.0005. **c** Luciferase reporter assay of enhancer activity for select JunB-bound regions at the *Il17a* locus, in WT^*CD4cre*^ and KO^*CD4cre*^ naive CD4^+^ T cells cultured under Th17 cell conditions for 48 h. The JunB-bound CNS of interest at the *Il17a* locus are indicated in **b**. *Error bars* represent SEM of two independent experiments. **d** Luciferase reporter assay of silencer activity, as in **b**. *Error bars* represent SEM of two independent experiments. **e** QPCR analysis of the expression of *Irf8* transcript in WT^*CD4cre*^ and KO^*CD4cre*^ 48 h Th17 cell polarization cultures, with *Irf8* ^*fl/fl*^CD4cre Th17 cells as a negative control. mRNA expression is presented relative to *Actb* expression. Data are representative of three independent experiments, with *error bars* representing SEM. **f** Flow cytometry of IRF8 expression in WT^*CD4cre*^ (*gray*), KO^*CD4cre*^ (*black*), and *Irf8* ^*fl/+*^KO^*CD4cre*^ (*teal*) Th17 cells 72 h post polarization, with *Irf8* ^*fl/fl*^ *CD4cre* (*shaded*) Th17 cells as a negative control. Data are representative of three independent experiments. **g** JunB ChIP-Seq and MACS2 analysis tracks at the *Irf8* locus, in 48 h polarized Th17 cells. **h** Flow cytometry of IL-17A production by WT^*CD4cre*^, KO^*CD4cre*^, and *Irf8* ^*fl/+*^KO^*CD4cre*^ 72 h Th17 cell polarization cultures. Relative percentages of IL-17A^+^ cells compared to WT^*CD4cre*^ cultures are displayed. *p<0.05; **p<0.01; ****p<0.0001 (unpaired two-tailed Student’s *t*-test)

To uncover JunB targets that stabilize effector identity, we performed RNA-Sequencing (RNA-Seq) and differential expression analysis of WT^*CD4cre*^ vs. KO^*CD4cre*^ Th17 cell cultures. Pathway analysis of the top differential genes identified those associated with Th cell differentiation as most significantly enriched (Supplementary Fig. 3a). Several Th-related genes are highlighted in the volcano plot displaying fold change in JunB-dependent transcripts (Fig. 3a). As KO^*CD4cre*^ cultures exhibit effector cell heterogeneity, we confirmed JunB regulation via global JunB occupancy determined by chromatin immunoprecipitation sequencing (ChIP-Seq). Of note, we identified Th17 cell effector genes—including *Il21*, *Il23r*, and *Il17a*—as direct JunB targets, being significantly reduced in gene expression in KO^*CD4cre*^ Th17 cell cultures (Fig. 3a), and displaying significant JunB locus-proximal occupancy (Fig. 3b and Supplmentary Fig. 3b). At these loci, JunB binding is associated with that of p300, a histone acetyltransferase for which occupancy is predictive of tissue-specfic enhancer activity^33^. Importantly, unlike other Th17 cell regulators, BATF^19^, IRF4^18^, and STAT3^17^, JunB is not required for *Rorc* (RORγt) expression (Fig. 3a). Thus, JunB directly activates key components of the Th17 cell program, independent of effects on *Rorc*.

We further explored JunB regulation of *Il17a*. Motif analysis of three JunB-occupied regions within the *Il17a* locus identified AP-1 consensus sites in each (Supplementary Table 1), implicating these conserved noncoding sequences (CNS) as putative JunB-dependent enhancers (Fig. 3b). Genomic regions corresponding to CN S+0.9,+6, and +10 were cloned upstream of a minimal promoter driving a luciferase reporter, and assayed for activity in both WT^*CD4cre*^ and KO^*CD4cre*^ in vitro differentiated Th17 cells. Only CNS+10, located 10 kb downstream of the *Il17a* transcription start site (TSS), displayed robust enhancer function, with an 8-fold increase in activity over that of the minimal promoter (Fig. 3c). Although CNS+10 activity was markedly less than that of CNS-5, an in vivo validated 5’ *Il17a* locus enhancer^34^, its activity was uniquely JunB-dependent, reduced by 2-fold in JunB-deficient Th17 cells (Fig. 3c). Notably, when test regions were assayed in *cis* with CNS-5, CNS+10 exclusively synergized with CNS-5 in a JunB-dependent manner (Fig. 3d), suggesting a mechanism for JunB-promoted maintainance of *Il17a*. The identification of CNS+10 as a novel JunB-dependent enhancer supports the view that JunB directly activates Th17 cell identity.

Among JunB-dependent genes, IRF8 is a compelling candidate mechanistic node for JunB-mediated Th17 cell stability. IRF8 is a negative regulator of Th17 cell differentiation^35^, and can replace IRF4 in BATF-JunB AP-1 complexes in Th17 cells^24^. *Irf8* RNA and protein levels were significantly elevated in KO^*CD4cre*^ Th17 cells and in KO*Il17a* ^ZsGreen-FM+^ cells (Fig. 3e, f and Supplementary Fig. 3c). Additionally, JunB binds upstream of the *Irf8* locus (Fig. 3g), suggesting that direct *Irf8* suppression contributes to stabilization of the Th17 cell program.

To assess whether IRF8 upregulation accounts for the decreased differentiation of JunB-deficient Th17 cells, we counteracted *Irf8* derepression by conditionally deleting one allele of *Irf8* on a KO^*CD4cre*^ background (generating *Irf8* ^f/+^ *Junb* ^f/f^ CD4-Cre mice), thereby restoring *Irf8* RNA and protein to near wild-type levels (Supplementary Fig. 3d and Fig. 3f). This coincided with a 50% rescue in IL-17A production (Fig. 3h); and a significant increase in the expression of lineage-relevant IRF8 repression targets (*Il23r*, *Il21*, *Ccr6*, and *Rorc*) in *Irf8* ^f/+^KO^*CD4cre*^ relative to KO^*CD4cre*^ Th17 cells (Supplementary Fig. 3e). Although heterozygous deletion of *Irf8* partially restored the Th17 cell program in the absence of *Junb*, it did not reverse the aberrant elevation of T-bet, IFNγ, and Foxp3 in KO^*CD4cre*^ Th17 cells (Supplementary Fig. 3f). Therefore, the release of alternative CD4^+^ T-cell programs upon loss of JunB is independent of IRF8 upregulation. Taken together, JunB promotes Th17 cell identity by both limiting the expression of IRF8 and directly activating effector loci.

### JunB directly represses alternative programs in Th17 cells

**Fig. 4:**
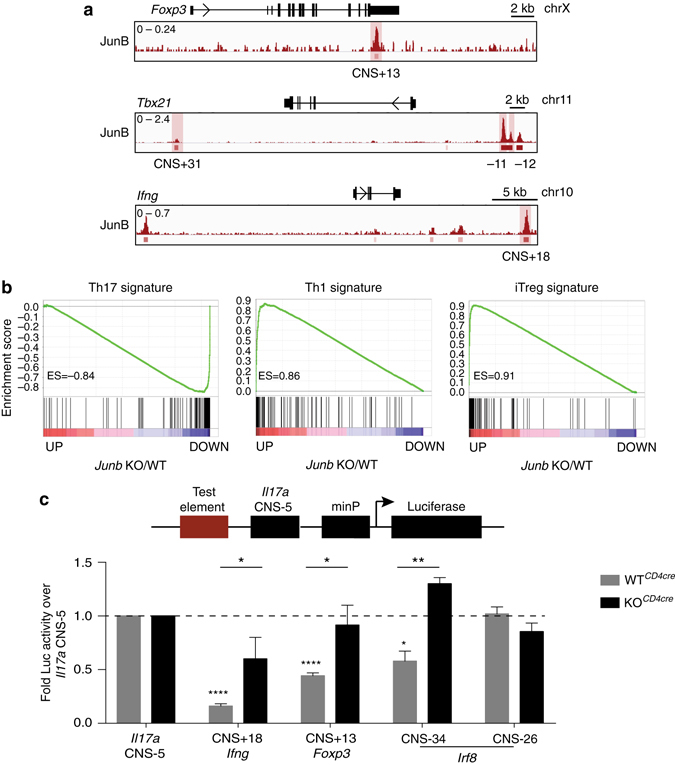
JunB binding defines novel silencer elements in Th17 cells. **a** ChIP-Seq tracks of JunB occupancy at the *Foxp3*, *Tbx21*, and *Ifng* loci in WT Th17 cells from 48 h polarization cultures. Lower track marks the boundaries of significant peaks called by MACS2 with FDR < 0.0005. **b** Gene set enrichment analysis (GSEA; Boad Institute) for KO^*CD4cre*^ relative to WT^*CD4cre*^ 48 h Th17 cell polarization cultures demonstrating significant enrichment of gene sets associated with Th1, iTreg and Th17 cell programs in the absence of *Junb* (*p* < 1 × 10^−4^). ES, enrichment score **c** Luciferase reporter assay of silencer activity for select JunB-bound regions at the *Ifng*, *Foxp3*, and *Irf8* loci, in WT^*CD4cre*^ and KO^*CD4cre*^ naive CD4^+^ T cells cultured under Th17 polarizing conditions for 48 h. JunB-bound CNS of interest at each locus are indicated in **a**. *Error bars* represent SEM of three independent experiments. *p<0.05; **p<0.01 (unpaired two-tailed Student’s *t*-test)

The lineage dysregulation observed for JunB-deficient Th17 cells was also apparent by global differential gene expression analysis (Fig. 3a). In particular, transcripts for subset-defining regulators of Treg and Th1 differentiation, including *Foxp3*, *Tbx21*, and *Ifng*, were significantly upregulated in KO^*CD4cre*^ Th17 cells (Fig. 3a and Supplementary Fig. 4a). This coincided with significant gene-proximal JunB ChIP occupancy (Fig. 4a), identifying these lineage-defining loci as likely direct JunB repression targets. Gene set enrichment analysis was used to characterize the dysregulated transcriptome of KO^*CD4cre*^ relative to WT^*CD4cre*^ Th17 cells (Fig. 4b). In the absence of JunB, downregulated targets were significantly enriched in Th17 cell signature genes, whereas upregulated genes were significantly enriched for those associated with Treg and Th1 cell signatures, indicating global shifts in effector programming consistent with the adoption of alternative CD4^+^ T-cell states.

### JunB occupancy defines novel silencer elements

Cell identity is governed by the interaction between environment-sensing TFs, chromatin context, and the DNA elements that regulate lineage-relevant loci. The *cis* elements that restrict alternative potentials in Th17 cells are undefined. To address this, putative JunB-dependent silencer elements were identified as JunB-occupied CNS proximal to lineage-defining repressed loci (*Irf8*, *Foxp3*, *Tbx21*, and *Ifng*), which also contained AP-1 consensus sites (Fig. 3g and Fig. 4a, Supplementary Table 1). Candidate regions were cloned upstream of the JunB-independent *Il17a* CNS-5 enhancer in a minimal promoter-driven luciferase reporter construct, and assayed in Th17 differentiation cultures for the ability to diminish enhancer activity. Three of seven regions displayed silencer activity, including *Ifng* CNS+18, *Foxp3* CNS+13 (3’UTR-localized), and *Irf8* CNS-34. Each significantly attenuated the activity of *Il17a* CNS-5 (Fig. 4c), and did not display enhancer activity (Supplementary Fig. 4b). Notably, when these 3 silencer CNSs were assayed in KO^*CD4cre*^ Th17 cells, *Il17a* CNS-5 activity was significantly restored and, in the case of *Irf8* CNS-34, enhanced (Fig. 4c), indicating that their repressive potential is JunB-dependent. Interestingly, none of the *Tbx21* CNS demonstrated silencing activity (Supplementary Fig. 4c), potentially reflecting the presence of distal *cis* elements^36^. Together, these data implicate *Ifng* CNS+18, *Foxp3* CNS+13, and *Irf8* CNS-34 as novel JunB-dependent silencer elements, which coordinate to protect Th17 cell status, and through regulation of *Ifng* and *Foxp3*, are potential restriction elements that actively repress alternative Th1/Treg cell fates.

### JunB deficiency results in enhanced JunD occupancy

**Fig. 5:**
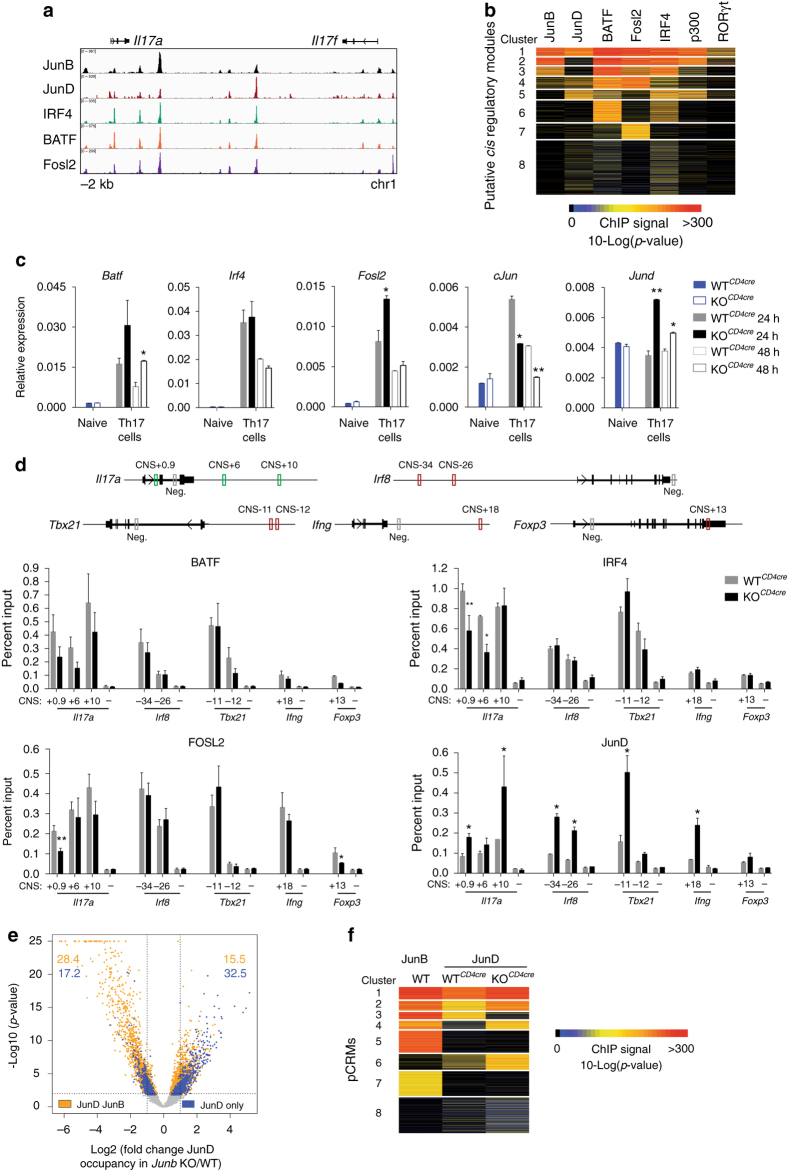
Enhanced JunD occupancy in the absence of JunB. **a** ChIP-Seq tracks for JunB, JunD, IRF4, BATF, and Fosl2 at the *Il17a* and *Il17f* loci in 48 h polarized wild-type Th17 cells. **b** Clustered heat map of pCRM regions of JunB, JunD, BATF, Fosl2, IRF4, p300, and RORγt occupancy in wild-type 48 h Th17 cell polarization cultures. **c** QPCR analysis for *Batf*, *Irf4*, *Fosl2*, *cJun*, and *Jund* transcripts, relative to *Actb* expression, in WT^*CD4cre*^ and KO^*CD4cre*^ CD4^+^ naive T cells and naive CD4^+^ T cells cultured under Th17 cell conditions for 24 and 48 h. **d** ChIP of WT^*CD4cre*^ and KO^*CD4cre*^ 48 h polarized Th17 cells, using α-BATF, α-IRF4, α-Fosl2, or α-JunD followed by QPCR. Primers used encompassed the AP-1 consensus site at select JunB-bound regions or a negative control region, as depicted with boxes in the schematics of the relevant loci. ChIP recovery at negative control loci provides local background for the assay. *Error bars* represent SEM of two independent experiments. **e** Differential global occupancy of JunD in KO^*CD4cre*^ vs. WT^*CD4cre*^ 48 h Th17 cell polarization cultures displayed as a volcano plot of fold change vs. significance. Differential binding of JunD is considered significant below FDR < 0.05; pCRMs with and without JunB co-occupancy displayed in *orange* and *blue*, respectively. The percentage of pCRMs for each subtype with fold change≥2 and *p*-value<0.01 are indicated for the regions bounded by *gray* trendlines. *p*-value capped at 10^−25^. **f** Clustered heat map of pCRM regions defined by occupancy of JunB and JunD in WT (C57Bl/6), WT^*CD4cre*^, or KO^*CD4cre*^ 48 h Th17 cell polarization cultures, as indicated. *p<0.05; **p<0.01 (unpaired two-tailed Student’s *t*-test)

JunB is a core component of AP-1 complexes in Th17 cells. As the main BATF heterodimerization partner^19^, JunB binds cooperatively to AP1-IRF composite motifs with BATF and IRF4^23^, essential pioneer factors for Th17 cell specification^14^. JunB also dimerizes with Fosl2, a negative regulator of effector potential in Th17 cells^14, 19, 24^. Consistent with this diversity, ChIP-Seq revealed a high degree of colocalization of JunB with known partners at Th17 cell (*Il17a*), and alternative Th subset (*Ifng*) loci (Fig. 5a and Supplementary Fig. 5a)^23^. To examine global patterns of TF binding in Th17 cells, we merged overlapping binding peaks for AP-1 TFs, IRF4, RORγt, and p300 into putative *cis* regulatory modules (pCRM), and generated a clustered heatmap of TF distribution at pCRMs. JunB showed a striking, near-exclusive binding overlap with BATF, Fosl2, and IRF4, indicating global occupancy of common regulatory regions (Fig. 5b, clusters 1, 2, and 3).

In light of the strong regulatory interaction between AP-1 TFs, we characterized how the loss of JunB influences the chromatin occupancy of JunB binding partners in Th17 cells. Importantly, *Batf*, *Irf4*, and *Fosl2* transcript levels were not reduced in KO^*CD4cre*^ relative to WT^*CD4cre*^ Th17 cell polarization cultures by 48 h (Fig. 5c). ChIP for BATF, IRF4, and Fosl2 from KO^*CD4cre*^ cultures demonstrated comparable occupancy to WT^*CD4cre*^ at select JunB-bound regions in the *Il17a*, *Irf8*, *Tbx21*, *Ifng*, and *Foxp3* loci (Fig. 5d). A notable reduction was observed for IRF4 at the *Il17a* locus, potentially reflecting replacement by elevated IRF8^24^ (Fig. 3f), which can bind to and regulate *Il17a* both directly and indirectly^35^. Therefore, the DNA binding pattern of Th17 cell-relevant AP-1 complex TFs is largely independent of JunB, unlike the collaborative binding of BATF with IRF4, which is greatly reduced in cells lacking the respective partner^14, 23, 24^.

The incomplete block in Th17 cell differentiation in the absence of JunB, compared to either BATF or IRF4, implies that other Jun family members play a compensatory role^23, 24^. JunD is an excellent candidate in this regard. Whereas *cJun* transcripts were dramatically reduced in KO^*CD4cre*^ relative to WT^*CD4cre*^ cultures by 48 h (Fig. 5c), *Jund* expression was elevated in *Junb*-deficient cells specifically under Th17-promoting conditions (Fig. 5c and Supplementary Fig. 5b). Moreover, 68% of significant genome-wide JunD binding peaks, vs. only 24% of cJun peaks, overlapped with those of JunB (Supplementary Fig. 5c and d). Like JunB, JunD can bind cooperatively to DNA with BATF and IRF4^23^. Accordingly, the global binding pattern of JunD in Th17 cells highly overlapped with that of BATF, and IRF4 as reported ^23^, and also with that of Fosl2 and JunB (Fig. 5a, b, cluster 1, 5).

The overlap in JunD and JunB occupancy suggests an antagonistic relationship. In addition to shared pCRMs (Fig. 5b, cluster 1), JunB binding in regions devoid of JunD implies that JunD is excluded in some contexts (cluster 2,3). Consistent with TF antagonism, in the absence of JunB, we observed a striking elevation in the binding of JunD at many AP-1 consensus sites typically bound by JunB in lineage-defining regulatory loci (Fig. 5d). Notably, global ChIP-Seq revealed elevated JunD binding in JunB-deficient Th17 cell cultures at numerous sites throughout the genome (Fig. 5e). Among these, there are regions that acquire novel JunD binding when JunB is eliminated (Supplementary Fig. 5d) indicative of factor antagonism, either through competition for DNA binding (Fig. 5f, JunB^+^ cluster 4), or for co-factors (Fig. 5f, JunB-devoid cluster 6). Importantly, JunD binding is not globally elevated, as an abundance of JunB-bound pCRMs displayed significantly diminished JunD occupancy in the absence of JunB (Fig. 5e), potentially reflecting binding of JunB-JunD heterodimers. Furthermore, unlike JunB, Batf, or Fosl2, JunD localizes near-exclusively to p300-occupied pCRMs (Fig. 5b, clusters 1,5), implying a dominant activating role in Th17 cells. Taken together, JunD can effectively replace JunB as an AP-1 dimerization partner at select genomic regions that include key effector loci, suggesting a potential mechanism for repression to activation switches during lineage conversion events mediated by loss of JunB.

### Global coordination of JunB with Th17 specification factors

**Fig. 6:**
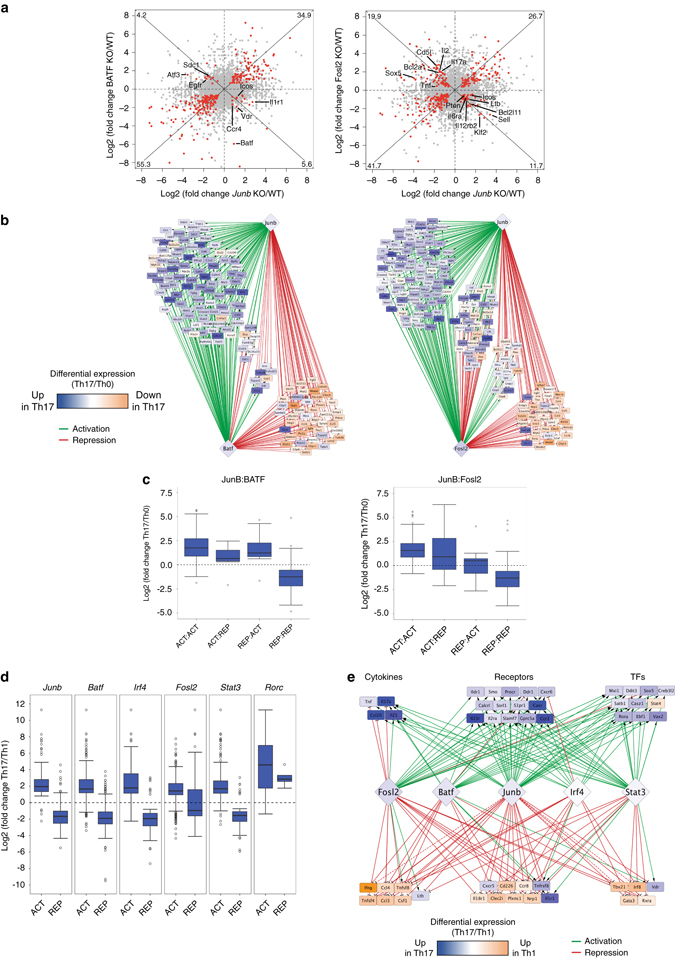
Global regulatory trends for targets of JunB, BATF, and Fosl2. **a** Comparison of differential expression (DE) targets of JunB in combination with BATF or Fosl2 from RNA-Seq of 48 h Th17 cell polarization cultures with individual TF KO. Genes highlighted in *red* show DE (FDR<0.05) in both datasets, and represent the subset of genes used for subsequent analyses. Numbers in corners indicate the percentage of all significant genes that fall within that quadrant. Genes with opposing AP-1 mediated regulation fall in quadrants 1 and 3, and select genes in these categories are labeled. **b** Network representation of shared targets of JunB and BATF or Fosl2. Networks were constructed by combining RNA-Seq and ChIP-seq data to determine direct regulatory targets. *Green* edges indicate activation, *red* edges indicate repression based on DE measured by RNA-Seq. Nodes are colored based on log2FC of DE in Th17 vs. Th0 RNA-Seq (only significantly DE nodes are included; FDR < 0.05), and are grouped based on combinatorial interactions between JunB and BATF or Fosl2 (i.e., activation:activation, activation:repression, etc.). **c** Comparison of the net effect of JunB:BATF and JunB:Fosl2 interactions on Th17-specific gene expression. Common targets were classified based on positive or negative regulatory action (ACT or REP) for each TF, then grouped based on 4 possible modes of cooperative regulation. The log2FC for DE genes (FDR<0.05) from Th17 vs. Th0 RNA-Seq is shown as a boxplot for each group. **d** Comparison of the Th17 vs. Th1 preference of activation and repression targets of Th17 TFs. Target genes were given regulatory classification as in **c**. For each category, the log2FC for genes with DE in Th17 vs. Th1 (FDR<0.05) is plotted. **e** Network representation of Th17 TF targets with functional categorization. Networks were constructed as in **b**, except with node coloring indicating log2FC of DE in Th17 vs. Th1 (FDR<0.05). Genes were classified and grouped based on molecular function. Only targets with JunB-connected edges are shown. *Dashed lines* indicated direct targets validated using ChIP-QPCR and luciferase assay that fall outside the 5 kb threshold used for global target classification

The interplay between AP-1 proteins in Th17 cells is complex as JunB can homodimerize, or form heterodimers with partners such as BATF or Fosl2. To examine global trends in the regulation of shared targets by individual AP-1 factors in Th17 cells, we compared the JunB-dependent differential expression profile with that of BATF and Fosl2 (Fig. 6a, *red dots*). Notably, the majority of TF-dependent genes (90%) were similarly affected (i.e. both up- or downregulated) in JunB- and BATF-deficient Th17 cells, in contrast to a comparison of JunB- and Fosl2-dependent genes, where a relatively large proportion (32%) demonstrate discordant regulation (Fig. 6a). Highly coordinate regulation of gene expression by JunB and BATF is consistent with the predominant formation of a heterodimer pair, vs. potentially greater JunB-independent diversity in AP-1 or co-factor associations for Fosl2.

To evaluate the regulatory collaboration between JunB and Th17 cell identity factors (BATF, Fosl2, IRF4 and STAT3), we defined direct, functional targets for each TF as those that are significantly differentially expressed in TF knockout vs. wild-type RNA-seq; and for which one or more significant TF ChIP-seq peaks localize within 5 kb of the gene. The high-confidence network of TF-target interactions are visualized in Cytoscape (Supplementary Fig. 6a). JunB target genes display a high degree of co-regulation by other Th17 cell specifying TFs, with 83% regulated by at least one additional TF. Notably, AP-1 TF subnetworks reveal a striking, near-absolute activation-repression concordance by JunB and BATF, with a lesser degree of coordinate regulation by JunB and Fosl2 (Fig. 6b), consistent with the comparisons of RNA differential expression (Fig. 6a). Coherent activation or repression activity by either JunB-BATF or JunB-Fosl2 collaborations are well correlated with actual changes in gene expression in Th17 relative to Th0 cells (i.e., genes activated by both TFs are upregulated in Th17 cells) supporting dominant regulatory action by AP-1 complexes in Th17 cell differentiation (Fig. 6b, c). Conversely, in instances where JunB and Fosl2 show opposing regulation, differential gene expression upon Th17 differentiation is most consistent with JunB activity, implicating JunB as a more influential activator and repressor (Fig. 6c).

Does JunB globally silence alternative CD4^+^ T-cell programs? Direct repression targets of JunB are significantly enriched for genes that are differentially expressed in Th1 vs. Th17 polarization cultures (Th1 signature in orange; Supplementary Fig. 6b and Fig. 6d), and similarly in iTreg vs. Th17 differentiation (Supplementary Fig. 6c), supporting a role for JunB in broad silencing of Th1- and iTreg-associated loci. This strong correlation is mirrored for repression targets of BATF, IRF4, and STAT3, but not of RORγt (Fig. 6d, Supplementary Fig. 6c). Consistent with a more general role in limiting effector plasticity, Fosl2 represses genes that are upregulated in Th1, iTreg, and Th17 signatures. Focusing our analysis on functional gene classes (e.g., cytokines, receptors, and TFs) reveals a high level of coordinated regulation of global Th17 (colored *blue*), Th1, and iTreg cell signature genes by JunB, BATF, IRF4, STAT3, and Fosl2 (Fig. 6e, and Supplementary Fig. 6d). The concerted negative regulation is highlighted for key Th1 and Treg loci (*Ifng*, *Tbx21*, *Il18r1*,*Tnfsf4*, *Nrp1*, and *Foxp3*) among others that are upregulated upon Th1 and iTreg differentiation. JunB is also highly integrated into the global Th17 cell activation program coordinated by initiator TFs BATF, IRF4 and STAT3^14^. Indeed, the Th17 cell signature is strongly enriched among direct JunB activation targets (Fig. 6d), and JunB co-activates many of these signature genes (*Il17a*, *Il23r*, *Ccl20*, and *Rora*) in conjunction with Th17 pioneer TFs (Fig. 6e). In addition to influencing ‘master’ regulator loci, JunB directly contributes more broadly to global regulatory programs that promote Th17 cell identity and restrict alternative Th1 and Treg programs.

### Steady-state Th17 cells are unaltered in the absence of JunB

**Fig. 7:**
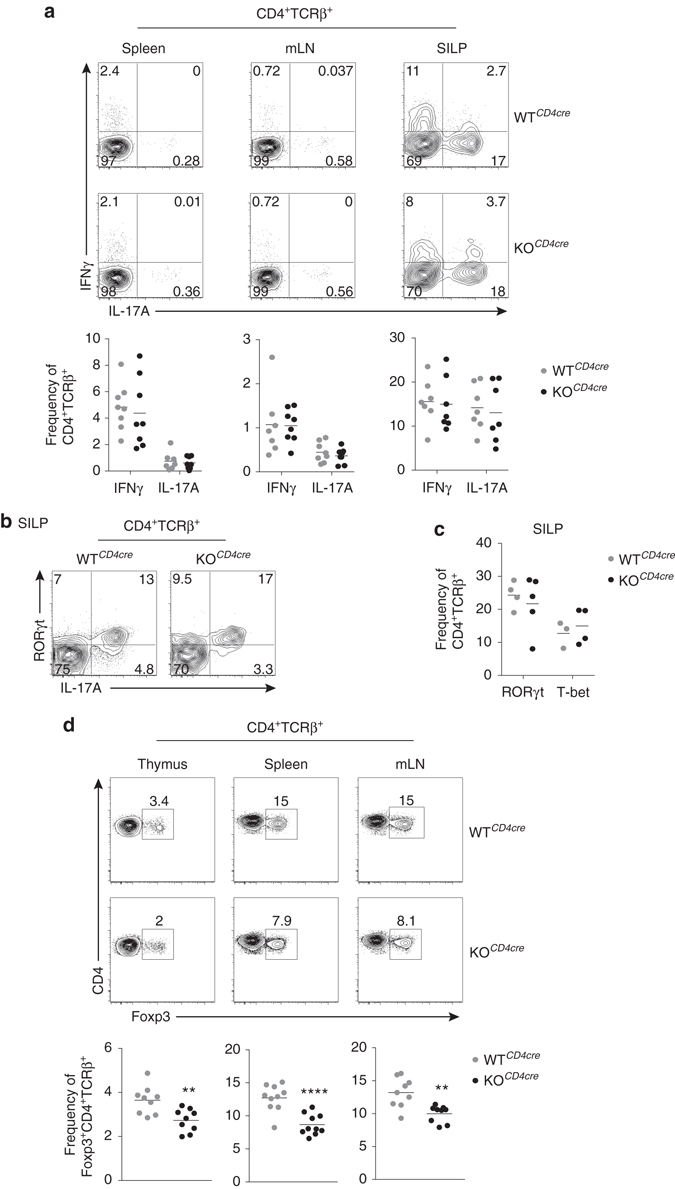
JunB is not required for homeostatic CD4^+^ Th cell differentiation. **a** Flow cytometry of intracellular IL-17A vs. IFNγ in CD4^+^TCRβ^+^ cells from the spleen, mesenteric lymph node (mLN), and small intestine lamina propria (SILP), of WT^*CD4cre*^ and KO^*CD4cre*^ mice. The frequencies of total IL-17A and IFNγ producing CD4^+^TCRβ^+^ cells from each tissue are shown. **b** Flow cytometry of RORγt and IL-17A expression in CD4^+^TCRβ^+^ cells from the SILP of WT^*CD4cre*^ and KO^*CD4cre*^ mice. **c** Frequencies of RORγt^+^ and T-bet^+^ CD4^+^TCRβ^+^ cells from the SILP of WT^*CD4cre*^ and KO^*CD4cre*^ mice are shown. **d** Flow cytometry of Foxp3 expression by CD4^+^TCRβ^+^ cells from the thymus, spleen, and mLN of WT^*CD4cre*^ and KO^*CD4cre*^ mice. Frequencies of Foxp3^+^CD4^+^TCRβ^+^ cells from each tissue are shown. **p<0.01; ****p<0.0001 (unpaired two-tailed Student’s *t*-test)

Given the dysregulation of JunB-deficient polarization cultures, we examined whether Th17 cell differentiation was also altered in vivo. Notably, there was no difference in the proportion of IL-17A- or IFNγ-expressing steady-state CD4^+^ T cells localized to the spleen, mesenteric LNs, or small intestine lamina propria (SILP) between WT^*CD4cre*^ and KO^*CD4cre*^ mice (Fig. 7a). The frequency of SILP CD4^+^ T cells expressing RORγt or T-bet was also unaltered in JunB mutants (Fig. 7b, c). However, there was a modest reduction in the frequency and number of Foxp3^+^ Treg cells in KO^*CD4cre*^ mice in every tissue evaluated (Fig. 7d and Supplementary Fig. 7a). The decrease in Treg cells in vivo is similar to the defect observed when IL-2 is omitted from mutant iTreg cultures in vitro (Supplementary Fig. 7b). This is in contrast to the unperturbed status of IL-2 supplemented cultures (Fig. 1c), suggesting a potential non cell-intrinsic role for JunB in Treg cell development or maintenance. Accordingly, deletion of *Junb* exclusively in Foxp3^+^ cells resulted is similar frequencies of Tregs compared to control mice in secondary lymphoid organs (Supplementary Fig. 7c). Overall, steady-state CD4^+^ Th cell subsets, including homeostatic Th17 cells, were similar in WT^*CD4cre*^ and KO^*CD4cre*^ mice.

### JunB promotes Th17 cell subset stability in vivo

**Fig. 8:**
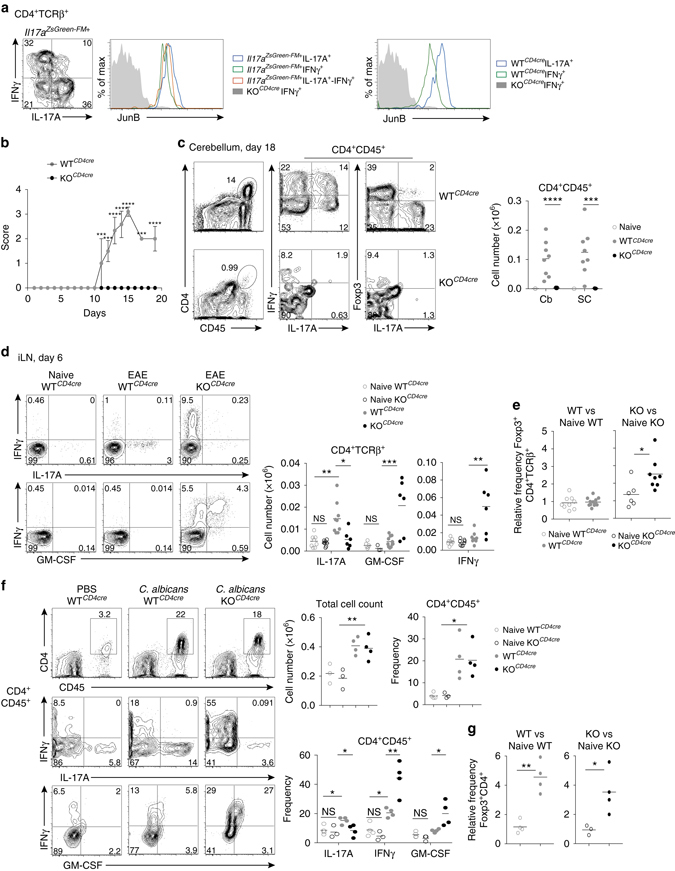
JunB promotes inflammatory Th17 cells and limits alternative CD4^+^ Th cell programs in vivo. **a** Flow cytometry of JunB expression by *Il17a* ^ZsGreen-FM+^CD4^+^TCRβ^+^ cells producing IL-17A, IFNγ, or IL-17A-IFNγ in the draining lymph node of WT^*Il17aCre*^ *R26* ^*ZS*^ mice day 6 post induction of EAE, with KO^*CD4cre*^ as a negative staining control (*left*). Flow cytometry of JunB expression in CD4^+^TCRβ^+^ IL-17A or IFNγ producing cells from the inguinal lymph node of WT^*CD4cre*^ mice, with KO^*CD4cre*^ cells as a negative staining control (*right*). Data are representative of two independent experiments. **b** WT^*CD4cre*^ and KO^*CD4cre*^ mice were injected with an emulsification of Complete Freund’s Adjuvant and MOG_35-55_ peptide and monitored for disease severity over time. Error bars represent SEM of six mice in each cohort, from three independent experiments. **c** Flow cytometry of IL-17A, IFNγ, and Foxp3 expression in CD4^+^CD45^+^ cells isolated from the cerebellum at day 18 post induction of EAE and restimulated ex vivo. Plots are representative of all mice analyzed in **a**. Total cell numbers of CD4^+^CD45^+^ cells in the cerebellum and spinal column of WT^*CD4cre*^ and KO^*CD4cre*^ mice, as well as naive control, when WT^*CD4cre*^ mice were at max score are shown. **d** Flow cytometry of cytokine production in WT^*CD4cre*^ and KO^*CD4cre*^ CD4^+^TCRβ^+^ cells from the draining lymph node of mice on day 6 post induction of EAE and restimulated ex vivo. Total cell numbers of CD4^+^TCRβ^+^ cells producing IL-17A, IFNγ, or GM-CSF are shown. ns, not significant. **e** Relative frequency of Foxp3^+^CD4^+^TCRβ^+^ cells in the draining lymph node of WT^*CD4cre*^ and KO^*CD4cre*^ mice at day 6 post-induction of EAE relative to naive mice of the same genotype. **f** *C. albicans*. cutaneous infection model in WT^*CD4cre*^ and KO^*CD4cre*^ mice. Flow cytometry of cytokine production by ex vivo restimulated CD4^+^CD45^+^ cells harvested from the skin 5 days post infection, as well as naive control. Data are compiled from three independent experiments. **g** Relative frequencies of Foxp3^+^CD4^+^CD45^+^ by ex vivo restimulated WT^*CD4cre*^ and *KO* ^*CD4cre*^ cells harvested from the skin 5 days post infection, compared to their respective naive controls. *p<0.05; **p<0.01; ***p<0.001; ****p<0.0001 (unpaired two tailed Student’s *t*-test)

To evaluate the role of JunB in restricting alternative CD4^+^ T-cell programs in an inflammatory setting where Th17 cell plasticity occurs physiologically, we used the EAE mouse model of MS, which is dependent upon the activity of CD4^+^ Th cells^37^. In EAE, IFNγ^+^ encephalitogenic T cells originate nearly entirely from Th17 cells that have undergone subset conversion^11^. Notably, JunB levels decrease as *Il17a* ^ZsGreen-FM+^ CD4^+^ T cells transition from IL-17A^+^ to IL-17A^+^IFNγ^+^ or to IFNγ^+^ status post EAE induction. Consistent with in vitro differentiation cultures (Fig. 1a), JunB is more highly expressed by IL-17A- vs. IFNγ-producing CD4^+^ T cells (Fig. 8a). These trends indicate that JunB protein levels are modulated during physiological Th17 cell plasticity, suggesting that JunB is part of the mechanism that regulates effector conversions. Thus, EAE is an ideal model to evaluate the role of JunB in Th17 cell lineage stability.

Following induction of EAE, WT^*CD4cre*^ mice progressed to near-complete paralysis, whereas KO^*CD4cre*^ mice were absolutely protected from disease (Fig. 8b). Analysis of cerebellum and spinal columns at peak disease in WT^*CD4cre*^ mice, uncovered a dramatic reduction in encephalitogenic CD4^+^CD45^+^ cells infiltrating the central nervous system (CNS) of KO^*CD4cre*^ mice, precluding cellular phenotyping (Fig. 8c). This likely accounted for the lack of symptoms, and revealed an unexpected requirement for JunB for pathogenic T-cell CNS infiltration. Accordingly, genes involved in migration are enriched in the altered transcriptome of JunB-deficient Th17 cells (Supplementary Fig.3a). To assess early CD4^+^ T-cell responses, draining LNs were examined at 6 days post induction of EAE, where there is no significant difference in baseline effector profiles in naive KO^*CD4cre*^ mice (Fig. 8d). This revealed a striking dysregulation in CD4^+^ T-cell cytokine production in KO^*CD4cre*^ mice similar to that of JunB-deficient Th17 cell cultures (Fig. 8d). In particular, KO^*CD4cre*^ mice had fewer IL-17A-producing CD4^+^ T cells compared to WT^*CD4cre*^ mice, with numbers similar to naive levels (Fig. 8d). Importantly, mutant CD4^+^ T cells were not refractory to activation; rather, KO^*CD4cre*^ LNs showed a striking predominance of IFNγ- and GM-CSF-producing CD4^+^ T cells relative to WT^*CD4cre*^ EAE mice (Fig. 8d). Enhanced GM-CSF was also a feature of KO^*CD4cre*^ Th17 cell cultures in vitro (Supplementary Fig. 8a). Moreover, taking into account that Treg cells are reduced in steady-state KO^*CD4cre*^ mice, we observed a selective twofold increase in the frequency of LN Foxp3^+^CD4^+^ T cells relative to naive mice at day 6 of EAE, implying that enhanced Treg induction also occurs in vivo in the absence of JunB (Fig. 8e and Supplementary Fig. 8b). Notably, while JunB is essential for inflammatory Th17 cell induction and expression of important effector genes, JunB does not globally regulate the previously defined pathogenic Th17 cell profile^30^ (Supplementary Fig. 8c). Thus, in an inflammatory setting, JunB is essential for the induction of appropriate Th17 cell responses; for restricting alternative CD4^+^ Th and Treg programs; and for enabling pathogenic CD4^+^ T-cell CNS migration during EAE.

To assess the role of JunB in regulating lineage conversion events during inflammation, we induced EAE in WT^*Il17aCre*^ *R26* ^*ZS*^ and KO^*Il17aCre*^ *R26* ^*ZS*^ mice and tracked fate-mapped *Il17a* ^ZsGreen-FM+^ cells in vivo. Similar to KO^*CD4cre*^ mice, *Junb* deletion in *Il17a*-expressing cells resulted in a complete rescue from symptoms (Supplementary Fig. 8d). At peak disease, 50% of CD4^+^ CNS infiltrating cells in WT^*Il17aCre*^ *R26* ^*ZS*^ mice were *Il17a* ^ZsGreen-FM+^ and enriched for encephalitogenic IL-17A^+^IFNγ^+^ CD4^+^ T cells, as reported^11^ (Supplementary Fig. 8e). In contrast, although CD4^+^ T cells were identified in the CNS in KO^*Il17aCre*^ *R26* ^*ZS*^ mice, these were selectively devoid of the *Il17a* ^ZsGreen-FM+^ fraction that marks JunB-deficient cells. Unexpectedly, this specific depletion was mirrored in the draining LN early post-induction of EAE in KO^*Il17aCre*^ *R26* ^*ZS*^ vs. WT^*Il17aCre*^ *R26* ^*ZS*^ mice (Supplementary Fig. 8f), and this coincided with a dramatic elevation in the frequency of AnnexinV^+^ apoptotic cells (Supplementary Fig. 8g). While the loss of JunB-deficient *Il17a* ^ZsGreen-FM+^ T cells precluded analysis of fate conversion events, it revealed a distinct requirement for sustained JunB expression for survival of differentiated Th17 cells. Importantly, an unaltered frequency of AnnexinV^+^ cells among homeostatic SILP Th17 cells suggests that the role of JunB in Th17 cell survival is restricted to an inflammatory setting (Supplementary Fig. 8h).

In addition to the chronic inflammatory context of EAE, we employed an acute cutaneous infection model with *Candida albicans* to assess the role of JunB in an inflammatory setting where Th17 cells do not typically exhibit plasticity^11^. IL-17A-producing Th17 cells are important in the defense against *C*. *albicans* ^38, 39^. On day 5 post infection, similar frequencies of CD4^+^ cells were observed in the skin explants from WT^*CD4cre*^ and KO^*CD4cre*^ mice, both significantly enhanced relative to mock-treated controls (Fig. 8f). As expected, WT^*CD4cre*^ mice produced a balanced IL-17A/IFNγ cytokine response to *C*. *albicans* ^11^. However, comparable to our findings in the EAE model, infected KO^*CD4cre*^ mice failed to induce a Th17 cell response, instead having a proportion of IL-17A^+^ cells ressembling uninfected mice (Fig. 8f). Moreover, T-cell loss of *Junb* skewed the CD4^+^ effector profile toward a predominantly Th1 phenotype, characterized by a striking enhancement in IFNγ and GM-CSF production. This dysregulation reflects an inflammation-dependent role for JunB in effector cell generation as steady-state cytokine-producing skin CD4^+^ cells are unaltered in KO^*CD4cre*^ mice (Fig. 8f). However, as in other tissues, there is a reduction in Foxp3^+^ Treg cells in the skin of naive KO^*CD4cre*^ mice that persists post infection (Supplementary Figs. 8i and 7d), despite a similar relative expansion in WT^*CD4cre*^ and KO^*CD4cre*^ mice (Fig. 8g). The lack of increased enhancement of Treg cells in mutants relative to controls may reflect the non-supportive environment for Treg cells in KO^*CD4cre*^ mice (Fig. 7d and Supplementary Fig. 7), which is perhaps selectively exacerbated in an acute inflammatory setting of *C. albicans* infection that is inherently non-permissive to plasticity.

Taken together, the in vivo findings identify an absolute and selective requirement for JunB in the induction and maintenance of Th17 effector cells during inflammation, in the context of both chronic autoimmunity and acute infection. Coordinately, JunB serves an essential role in constraining Th1 and Treg features, suggesting that the modulation of JunB in an environment-dependent manner can permit controlled adoption of alternative effector programs.

## Discussion

Previous studies have identified the pioneer factors and transcriptional regulators required to implement the Th17 cell program downstream of TCR and cytokine signals, however, the mechanisms underlying the inherent plasticity of the Th17 cell subset are less characterized^14, 17–19^. Here, we define JunB as a novel, nonredundant regulator of global programs that support Th17 cell identity and that restrain alternative Th1 and Treg cell fates in inflammatory contexts.

Th17 cells are functionally heterogeneous. Those produced at steady-state promote intestinal barrier homeostasis and defense^4^, whereas those elicited by IL-23 exposure are pathogenic, express IFNγ and GM-CSF, and exacerbate inflammatory disease^10, 11, 40, 41^. Although all subsets are RORγt-dependent^16^, the TFs that distinguish the development of homeostatic vs. inflammatory Th17 cells are less defined. In this regard, JunB is a selective, obligatory regulator of inflammatory Th17 cell induction, but dispensable at steady-state. In contrast to Blimp-1 and RBPJ, which confer IL-23R-driven pathogenicity to Th17 cells^42, 43^, JunB restrains pathogenic IFNγ and GM-CSF production, and supports the underlying Th17 cell program. However, the selective role of JunB during inflammation does not discriminate pathogenic vs. non-pathogenic Th17 cell differentiation. Rather, JunB is necessary in the context of IL-6. JunB is essential for Th17 cell induction in the IL-6-dependent inflammatory contexts of EAE and *C*. *albicans* infection^44, 45^, but not for homeostatic IL-6-independent intestinal Th17 cell development^46^. In addition, JunB expression is upregulated by TCR activation in vitro, suggesting that the priming environment may drive unique inflammatory vs. homeostatic Th17 programs, whereby JunB differentially integrates into AP-1 complexes that program the regulatory landscape^14^.

JunB is required at distinct stages of inflammatory Th17 cell differentiation. It is necessary during initiation for robust induction of the Th17 cell effector program, and restraint of Th1 and Treg cell potentials. Thereafter, sustained JunB expression is essential to maintain identity and limit plasticity in differentiated Th17 cells. Examination of lineage conversion events in vivo were precluded by apoptosis following JunB withdrawal from *Il17a*-expressing cells. Therefore, once established, continued JunB activity supports Th17 cell viability in inflammatory environments. Notably, no CD4^+^ T-cell subset completely lacks JunB expression. Thus, whereas JunB deletion causes apoptosis, reduced levels of JunB, as observed in EAE for non-Th17 or ex-Th17 converted subsets, appear to be compatible with lineage conversion events. A similar modulation of JunB may contribute to Th17 cell transdifferentiation into pathogenic IFNγ^+^ex-Th17 cells during colitis, or into regulatory cells during resolution of inflammation^6, 13^. Lastly, JunB is obligatory for the infiltration of inflammatory CD4^+^ T cells into the CNS during EAE, but not the skin in *C. albicans* infection, impling that JunB regulates a factor that mediates CNS-specific entry. Taken together, JunB plays multiple essential roles in CD4^+^ T cells.

AP-1 complexes are fundamental to Th17 cell identity. Like JunB, Fosl2 modulates plasticity in Th17 cells^14^. Analysis of the collaboration between JunB and Fosl2 revealed both concordant and discordant regulation of shared target genes, highlighting shared and unique roles in Th17 cells. Indeed, both TFs restrain Th1 and Treg potential, however, whereas JunB predominantly activates the Th17 program, Fosl2 represses many Th17-relevant genes^14^. Conversely, the highly coincident gene regulation by JunB and BATF identifies BATF as the dominant regulatory partner for JunB in Th17 cells. Further, JunB is integrated into a broad collaboration with pioneer TFs, BATF and IRF4^14^. This encompasses both coordinate activation of Th17 lineage genes and repression of alternative Th1/Treg programs. The latter implies an unappreciated role for BATF and IRF4 in limiting alternative CD4^+^ T-cell potentials, which is otherwise masked by their absolute requirement during Th17 cell differentiation^18, 19^. Thus, it is tempting to speculate that BATF and IRF4 establish a general CD4^+^ T-cell enhancer landscape downstream of the TCR that is poised to respond to inductive cytokine signals, and exploited by various AP-1 complexes to facilitate effector switches.

Alterations in AP-1 complex composition coincide with CD4^+^ T-cell effector flexibility. In the absence of JunB, the binding of BATF, IRF4, and Fosl2 are largely unaltered at effector loci, whereas JunD binding is significantly elevated, suggesting that JunD replaces JunB in AP-1 complexes and alters regulatory dynamics. Accordingly, this coincides with a repression-to-activation switch at Th1- and Treg-promoting loci, suggesting antagonistic roles for JunB and JunD, consistent with their regulation of *Il4* expression in Th2 cells^47^. Moreover, TFs such as BACH2, the global repressor of CD4^+^ T-cell effector programs^48^, may collaborate with JunB in restricting alternative fates. Indeed, in CD8^+^ T cells, BACH2 impedes access of JunD to enhancers of terminal differentiation effector loci and prevents their aberrant activation^49^. A similar mechanism may regulate JunD function in Th17 cells, where extensive colocalization with p300 implies a dominant activating role for JunD. However, at Th17-relevant loci (e.g., *Il17a*), which are downregulated in JunB deficiency, JunD activity may be countered by the concomitant derepression of the Th17 cell negative regulator, IRF8^35^. The capacity of IRF8 to replace IRF4 in AP-1 complexes suggests a mechanism for the downregulation of *Il17a* and other Th17 cell-defining loci repressed by IRF8. Altogether, this supports the model that dynamic changes in AP-1 complex and co-regulator composition coordinates the loss of Th17 cell effector function with the acquisition of alternative fates during lineage conversion.

Silencers are conserved elements that permit context-dependent expression of target loci. Capitalizing on the role of JunB as a plasticity regulator, we identified novel silencers at *Ifng*, *Foxp3*, and *Irf8* where negative regulation by JunB is central to maintenance of Th17 cell effector status. It is tempting to speculate that the coordinated loss of JunB activity at a network of such silencers facilitates transdifferentiation by simultaneously reversing the restriction of alternative potentials (e.g., via *Foxp3*, and *Ifng*) and releasing antagonists of Th17 cell identity (e.g., *Irf8*). Although there are examples of silencers that enforce long-term lineage identity (e.g., *Cd4* ^50^, and *Zbtb7b* ^51, 52^), those elements that underlie plasticity by permitting flexible changes in expression between CD4^+^ T-cell states are undefined. An interesting candidate is *Ifng* CNS-18, implicated as an enhancer in Th1 cells^53^, and here, as a potent silencer in Th17 cells. Such dual-activity elements have been described^54, 55^. Encompassing activating and repressive functions in single *cis* elements proposes a mechanism for efficient toggling between effector states in changing environments.

The mechanisms governing Th17 cell plasticity in immunity and autoimmunity are unclear. Here, we identify JunB as an obligatory regulator of Th17 cell stability that promotes the development of inflammatory Th17 cells and restricts flexibility towards alternative effector and regulatory programs. Targetted modulation of JunB to attenuate conversion to pathogenic Th17 cell subtypes, or rather, to promote transdifferentiation to anti-inflammatory regulatory cells presents possible avenues for therapeutic intervention in disease.

## Methods

### Mice

Mice encoding a conditional *Junb* floxed allele were obtained from Erwin Wagner (CNIO, Spain), and backcrossed at least five generations onto the C57BL/6 background. *Junb* ^*fl/fl*^ mice were bred with CD4cre^29^ and *Il17a* ^*Cre*^deleter strains^29, 11^, allowing for pan T-cell deletion or Th17 cell deletion, respectively (Jackson Laboratory). To enable tracking of *Il17a* ^*Cre*^ activity, mice encoding a *loxP-STOP-loxP-ZsGreen* conditional allele in the ubiquitous *Rosa26* locus^32^ (Jackson Laboratory), were bred to *Junb* ^*fl/fl*^ mice in order to obtain *Junb* ^*fl/fl*^ *Il17a* ^*Cre+/*−^ *R26* ^*ZS+/*−^ mice. *Junb* ^*fl/fl*^CD4cre mice were also mated with mice harboring a conditional *Irf8* floxed allele, to generate *Junb* ^*fl/fl*^ *Irf8* ^*fl/+*^CD4cre mice (Jackson Laboratory). For deletion in Treg cells, *Junb* ^*fl/fl*^ mice were bred with *Foxp3* ^YFP-Cre^ mice (Jackson Laboratory). C57Bl/6 wild-type mice were obtained from Taconic Biosciences. Experimental mice were between 7 and 12 weeks of age, with no preference to gender. Sample size was chosen using Mead’s equation to ensure adequate power. No blinding or randomization was used. Mice were maintained in specific-pathogen-free conditions at Duke University and all experiments were performed with permission from and in accordance with the guidelines of the Duke Institutional Animal Care and Use Committee.

### Cell culture

Naive CD4^+^ T cells were sort-purified from pooled spleen and lymph nodes, using the MoFlo XDP (Beckman Coulter). In short, red blood cells were removed by incubation with ACK lysis buffer (Lonza), and the resultant single cell suspensions enriched for CD4^+^ T cells using depletion-based magnetic separation (MagniSort Mouse CD4 T Cell Kit; eBioscience; 8804-6821-74), in accordance with the manufacturer’s protocol. Thereafter, cells were surface stained with antibodies specific for CD4, CD25, CD44, and CD62L, and sorted to isolate CD4^+^CD25^−^CD44^+/lo^CD62L^+^ naive T cells. The sort-purified naive CD4^+^ T cells were cultured in 12-, 24-, or 48-well plates coated with anti-hamster IgG secondary antibody (MP Biomedicals), in IMDM supplemented with 10% FBS, penicillin (10 U/ml), streptomycin (10 μg/ml), glutamine (2 mM), gentamicin (50 μg/ml), and β-mercaptoethanol (55 μM) (complete IMDM). For TCR stimulation, cultures were provided with anti-CD3ε (145^−^2C11; 0.25 μg/ml) and anti-CD28 (37.51; 1 μg/ml). Cultures were also supplemented with anti-IL-4 (1B11; 2 μg/ml) and anti-IFNγ (XMG1.2; 2 μg/ml), in addition to IL-2 (50 U/ml) for Th0 cell conditions; IL-6 (1.25 ng/ml) and TGFβ1 (0.3 ng/ml) for Th17 cell conditions; IL-6 (1.25 ng/ml), IL-1β (20 ng/ml), and IL-23 (25 ng/ml) for pathogenic Th17 cell conditions; or TGFβ1 (5 ng/ml) and IL-2 (50 U/ml) for iTreg conditions. In Th1 cell polarizing conditions, cultures were supplemented with anti-IL-4 (2 μg/ml) and IL-12 (10 μg/ml), while Th2 cell cultures included anti-IFNγ (2 μg/ml) and IL-4 (2 ng/ml). After 72 h, Th1 and Th2 cell cultures were removed from TCR stimulation and supplemented with IL-2 (50 U/ml), while Th0, Th17, and iTreg cell cultures continued to receive TCR stimulation. If maintained past 72 h, blocking antibodies were omitted from all five culture conditions upon passaging. The WT^*Il17aCre*^ *R26* ^*ZS*^ and KO^*Il17aCre*^ *R26* ^*ZS*^ starter Th17 cell culture conditions used to assess lineage conversion, were the same as above, as were the Th17, Th0, iTreg, and Th1 cell conditions used to plate the sort-purified *Il17a* ^*ZsGreen-FM+*^ cells. All antibodies and cytokines were purchased from eBioscience unless otherwise indicated.

### Isolation of cells from tissues

Single cell suspensions of the thymus, spleen, and lymph nodes were obtained by mechanical disruption of the tissues through a 40 μm cell strainer (Falcon). To isolate leuckocytes from the small intestine lamina propria (SILP), the small intestine was harvested, fat removed, Peyer’s patches excised, and luminal contents expelled. The intestine was cut into 1–2 cm pieces and shaken in PBS containing 1 mM DTT to remove mucus. The intestinal pieces were then transferred to PBS supplemented with 5 mM EDTA and 10 mM Hepes, and incubated twice at 37 °C for 10 min with rotation, to remove the epithelial cell layer. After the second incubation, intestinal pieces were collected and washed with HBSS. Pieces were transferred to HBSS supplemented with collagenase D (1 mg/ml; Roche), DNaseI (0.1 mg/ml; Sigma), and dispase (0.1 U/ml; Worthington), and subsequently, minced with scissors and incubated in the digestion cocktail at 37 °C for 25 min with rotation. The intestinal suspension was passed through a 100 μm cell strainer and a discontinuous 40:80 Percoll gradient used to further remove epithelial cells and debris. Percoll gradient separation was performed by centrifugation at 2200 rpm for 22 min at room temperature, with no brake. SILP lymphocytes were collected at the Percoll interphase, washed, and resuspended in complete IMDM, followed by stimulation and flow cytrometic analysis as detailed above.

### Antibodies and flow cytometry

To analyze cytokine production, cultured or primary cells were incubated in complete IMDM with phorbol 12-myristate 13-acetate (PMA; 50 ng/ml; Sigma) and ionomycin (500 ng/ml; Sigma), in the presence of GolgiStop (BD) for 4 h, in a 37 °C tissue culture incubator. Single cell suspensions were surface stained with flourochrome-conjugated antibodies in PBS containing 0.5% BSA and 2 mM EDTA (staining buffer) for 30 min at 4 °C. Primary cells were blocked with anti-FcγR (93; Biolegend), prior to staining surface markers. To allow for the exclusion of dead cells in all analyses, a fixable viability dye (eBioscience) was included with the surface staining. For live cell flow cytometric analysis or cell sorting, cells were washed twice and resuspended in staining buffer. For intracellular staining, cells were washed twice with staining buffer and resuspended in Fixation-Permeabilization solution (Foxp3/Transcription Factor Staining Buffer Set; eBioscience; 00-5523-00), with subsequent intracellular staining performed as detailed in the protocol provided with the kit. For surface staining, antibodies were purchased from eBioscience against the following antigens: CD4 (RM4-5; 1 : 500), CD8α (53-6.7: 1 : 500), TCRβ (H57-597; 1 : 500), CD69 (H1.2F3; 1 : 200), and CD45 (30-F11; 1 : 500). Antibodies were also purchased from eBioscience for intracellular staining of the following antigens: IL-17A (eBio17B7; 1 : 400), IFNγ (XMG1.2; 1 : 400), Foxp3 (FJK-16S; 1 : 200), T-bet (eBio4B10; 1 : 300), GM-CSF (MP1-22E9; 1 : 200), IRF8 (V3GYWCH; 1 : 200), RORγt (B2D; 1 : 100), and IL-4 (11B11; BD; 1 : 100). For detection of JunB, an unconjugated primary antibody (C-11; sc-8051x; 1 : 2000) from Santa Cruz was used in combination with a fluorescence-labeled secondary anti-mouse IgG (M1-14D12; 1 : 600). AnnexinV staining (Annexin V Apoptosis Detection Kit; eBioscience; 88-8006-74) was performed prior to fixation and in accordance with the manufacturer’s protocol provided. For analysis of cell proliferation, cells were labeled with CFSE (Cell Trace CFSE Cell Poliferation Kit; Life Technologies; C34554) according to the protocol provided by the manufacturer and cultured in Th17 cell polarizing conditions for 72 h. All antibodies were purchased from eBioscience unless otherwise indicated. Cells were acquired on a BD FACSCantoII and then analyzed with FlowJo software (Tree Star).

### Western blot

For immunoblot of JunB, WT^*CD4cre*^ naive CD4^+^ T cells were cultured for 5 days in Th17, iTreg, Th1, or Th2 polarizing conditions. KO^*CD4cre*^ naive CD4^+^ T cells were also cultured in Th17 cells conditions as a negative control. On day 5, cells were harvested, resuspended in RIPA lysis buffer (25 mM Tris, 150 mM Nacl, 1% NP-40, 1% sodium deoxycholate, and 0.1% SDS), and incubated at 4 °C for 30 min with rotation. Lysates were sonicated and subsequently centrifuged to remove debris. Equivalent masses of protein were mixed with LDS sample buffer (Invitrogen), separated on a 10% acrylamide gel, and transferred to PVDF membrane. Membranes were blocked with TBST containing 5% nonfat dry milk, and subsequently, incubated with anti-JunB (N-17; sc-46x; 1 : 2000) or anti-Actin Ab-5 (C4/actin; BD Transduction laboratories; 1 : 2000) as a loading control. ECL western blotting substrate (Thermo Fisher Scientific) was used in accordance with the manufacurer’s instructions, for detection of horseradishperioxidase labeled secondary antibodies.

### Luciferase assay

Select JunB-bound regions within the *Il17a*, *Irf8*, *Tbx21*, *Ifng*, and *Foxp3* loci were assessed by dual luciferease reporter assays for enhancer and silencer activity by cloning the sequences upstream of a minimal promoter driving a luciferase gene (pGL4.21[luc2/minP]) or a characterized enhancer from the Il17a locus (CNS-5) cloned into pGL4minP, respectively^34^. Primers used for cloning are available in Supplementary Table 3. Sort-purified naive CD4^+^ T cells were cultured for 48 h in Th17 cell polarizing conditions (20 ng/ml IL-6 and 0.3 ng/ml TGFβ), and subsequently, harvested for electroporation. In short, 2.5−5 × 10^6^ cells were incubated on ice in 500 μl RPMI with 2 μg pCMVRL and 10 μg pGL4 construct, empty or encoding a JunB-bound region. Cells were electroporated using a BioRad GenePulser II at 325 V and 960 μF, and subsequently, placed back on ice for 10 min to recover. Cells were transferred to prewarmed Th17 cell culture medium, and harvested 20 to 24 h later. Luciferase assays were performed using the Dual Luciferase Reporter Assay System (Promega; E1910). For each sample, firefly luciferase measurements were normalized to renilla luciferase readings and data presented as fold change over empty pGL4minP or CNS-5-pGL4minP, as appropriate.

### Chromatin immunoprecipitation

Naive CD4^+^ T cells were sorted as CD25^−^CD44^lo/−^CD62L^+^ and cultured under Th17 cell polarizing conditions (20 ng/ml IL-6 and 0.3 ng/ml TGFβ) for 48 h. For each ChIP, 8 million Th17 cells were cross-linked with 1% paraformaldehyde for 10 min at room temperature. Chromatin was isolated by sequential resuspension in Farnham Lysis and RIPA buffers, and subsequently, fragmented with a Vibra-Cell VCX130PB (Sonics & Materials). For immunoprecipitation, anti-mouse IgG1 or protein G Dynabeads were incubated overnight at 4 °C with 10 μg anti-JunB (N-17; sc-46x), anti-JunB (C-11; sc-8051x), anti-BATF (WW8; sc-100974), anti-IRF4 (M-17; sc-6059x), anti-Fra2 (Q-20; sc-604x), or anti-JunD (329; sc-74x). The labeled beads were subsequently washed and incubated overnight with the sonicated chromatin preparation at 4 °C. After immunoprecipitation, the protein-DNA crosslinks were reversed by heating at 65 °C, followed by proteinase K and RNase A treatment. DNA was purified by extraction with Phenol/Chloroform/Isoamyl Alcohol and amplified by QPCR with primers specific for JunB-bound regions of interest at the *Il17a*, *Irf8*, *Tbx21*, *Ifng*, *Foxp3*, and *Il23r* loci, as well as an irrelevant region within each locus. Biological replicates were performed for each ChIP. Primers used can be found in Supplementary Table 4.

### Quantitative real-time PCR

RNA was extracted with Trizol, from sort-purified naive CD4^+^ T cells or naive CD4^+^ T cells cultured under Th0, Th17, iTreg, Th1, or Th2 cell conditions. The RNA was reverse transcribed using SSRTIV (Invitrogen; 18091050) according to the manufacturer’s protocol. The resultant cDNA served as the template for amplification of genes of interest and housekeeping gene (*Actb*), by QPCR using iQ SYBR Green Supermix (Bio-Rad Laboratories). Primers used can be found in Supplementary Table 2. Relative expression was calculated using the ΔCT method.

### EAE induction

To induce EAE, mice were injected subcutaneously at three locations on day 0 with 200 μg MOG_35-55_ peptide (United Biosystems) emulsified in CFA supplemented with 2 mg/ml heat-killed *Mycobacterium tuberculosis* (VGD, Inc./Voigt Global). On days 0 and 2, mice were also injected intraperitoneally with 200 ng of pertussis toxin (List Biologicals). Mice were assessed daily for symptoms and scores were assigned as follows: 0, no symptoms; 0.5, limp tail tip; 1.0, limp tail; 1.5, quick recovery after inversion; 2.0, cannot recover after inversion; 2.5, one weak/partially paralyzed hind leg; 3.0, two weak/partially paralyzed hind legs; 3.5, complete hind and partial front paralysis; 4.0, complete hind and front paralysis. Mice that reached score 4.0 were deemed moribund and euthanized. Leukocytes from the cerebellum and spinal column of mice with a score of 2.5 to 3.5 were harvested for phenotyping by flow cytometry. Briefly, mice were perfused with PBS containing 5 mM EDTA. The cerebellum was removed and placed in PBS supplemented with 1% FBS and 1 mM Hepes (PFH). The spinal column was expelled with PFH, using a 21 G needle affixed to a 20 mL syringe, and also placed in PFH. Following mincing, samples were digested in PFH containing 10 mg/ml collagenase D for 30 min in a 37 °C tissue culture incubator, and EDTA added to a final concentration of 12.5 mM to terminate the digestion. The digests were subsequently aspirated through a 3 ml syringe with 18 G needle and filtered through a 100 μm cell strainer. Cells were washed once with PFH, resuspended in 38% Percoll solution, and pelleted at 2000 r.p.m. for 30 min at RT with no brake. The cell pellet was washed once with PFH and resuspended in complete IMDM, followed by stimulation and flow cytrometic analysis as detailed above.

### Cutaneous infection with *Candida albicans*

*C. albicans* strain SC5314 was kindly provided by the laboratory of Dr. Joseph Heitman. They were grown in YPD medium at 30 °C overnight for yeast morphology, and the next day diluted to OD_600_ of 0.6 and cultured until OD_600_ reached 1.5^44^. The *C. albicans* was washed once with PBS, and resuspended to infect each mouse with 2 × 10^8^ yeast in 50 μl PBS. An ~2 cm^2^ region of the ventral skin was plucked and scotch tape used to abraize the region. The 50 μl *C. albicans* suspension was subsequently applied to the skin. On day 5 post infection, mice were euthanized and leukocytes isolated from the infected skin for phenotyping by flow cytometry. Briefly, the infected skin was excised and minced in complete IMDM. The tissue was digested for 1 h at 37 °C in complete IMDM supplemented with Collagenase D (1 mg/ml), dispase (5 U/ml), and DNase I (0.1 mg/ml). The digests were subsequently filtered through a 100 μm cell strainer and the cells washed once with PBS. The cells were then resuspended in 38% Percoll solution, and pelleted at 2000 r.p.m. for 30 min at RT with no brake. The cell pellet was washed once with PBS and resuspended in complete IMDM, followed by stimulation and flow cytrometic analysis as detailed above.

### Statistical analysis

A standard unpaired two tailed Student’s *t* test was used, unless otherwise stated, to determine the statistical probability of the difference observed between two populations of cells in our phenotyping analyses (GraphPad Software): *p<0.05; **p<0.01; ***p<0.001; ****p<0.0001. Parametric tests were used when variance between groups was similar, as determined by F-tests; however, when variance was significantly different non-parametric tests were performed.

### Library preparation and sequencing

RNA was purified from Th cultures for RNA-Seq using the Trizol method and used directly for library preparation. For ChIP-Seq, the same general ChIP protocol (see above) was used with some modifications. Briefly, 4 × 10^7^ Th17 cells were cross-linked and the resulting sonicate was subjected to immunoprecipitation using 10 μg of anti-JunB (N-17; sc-46x), anti-JunB (C-11; sc-8051x), or anti-JunD (329; sc-74x). Following reverse-crosslinking, ChIP and input samples were processed to obtain purified DNA as described^56^.

Raw material (RNA or DNA) was provided to the Duke Center for Genomic and Computational Biology for library preparation and sequencing. Sample integrity was first verified for quality using an Agilent Bioanalzyer. Libraries were then prepared for RNA-Seq using a Kapa Stranded mRNA-Seq Kit (Kapa Biosystems; Cat # KK8420), or for ChIP-Seq using a Kapa Hyper Prep Kit (Kapa Biosystems; Cat # KK8504). Libraries were then run on an Illumina HiSeq 2500 to generate 50 bp single-end reads. Sequence read quality was subsequently verified using FastQC, and only high-quality samples were used for downstream analysis.

### Sequence read alignment

Raw sequence data was either generated in house (*Junb* KO RNA-Seq and JunB ChIP-Seq) or downloaded from the NCBI SRA database (see Supplementary Data 1 for full listing of datasets used). For RNA-Seq experiments, raw sequence reads were aligned to the *Mus musculus* mm10 genome using the gapped-read alignment software Tophat2 (ver. 2.0.13), with the following parameters: “-a 10 -g 20—no-novel-juncs -G”^57^ using UCSC mm10 genes as a transcriptome reference. For ChIP-Seq experiments, raw sequence reads were aligned to the *Mus musculus* mm10 genome using Bowtie2 (ver. 2.2.5), with the setting “-k 1”^58^.

### Differential expression analysis

Reads were assigned to features found in the UCSC mm10 genome annotation and counted using the “count” function from the HTseq package (ver. 0.6.1), with options “-s no -m union”^59^. Raw feature counts were then analyzed for differential expression using the edgeR (ver. 3.16) software package^60^. Briefly, within each comparison, raw counts were filtered to remove genes with less than 1 count per million reads in at least two of the samples analyzed. Filtered counts for biological replicates were then used to sequentially estimate library size and dispersion by calling the estimateSizeFactors(), estimateCommonDisp(), and estimateTagwiseDisp() functions. Finally, differential expression testing was performed on normalized counts using the exactTest() function to generate a differential expression table, including log2 fold-change and FDR-adjusted *p*-values (Benjamini-Hochberg method) used in subsequent analyses. Genes were considered significantly different if having an FDR-adjusted *p*-value<0.05. Two independent biological samples were used for analysis.

### Peak calling, annotation, and visualization

Aligned ChIP-Seq reads were used for peak calling with MACS2 (ver. 2.1.0.20150731) with settings “-g mm -q 0.0005 -m 15 50—bw = 200”^61^. Called peaks were then mapped to genes using the “map_chip_peaks” script from the scorchR package, with scripts updated to accept MACS2 input^14^. Peaks were considered “associated” with a given gene if their summits fell within 5 kb up or downstream of the gene body, the boundaries of which were determined by transcription start and end sites. Putative *cis* regulatory modules were identified from combined MACS outputs and binned into 8-clusters for visualization as a heatmap using scorchR^14^. For the visualization of ChIP-Seq tracks, igvtools (Broad Institute) was used to generate TDF visualization files using the parameters “count -z 10 -w 10 -f mean”, which were subsequently viewed in the Integrative Genomics Viewer (IGV; Broad Institute). Two independent biological samples were analyzed producing similar results.

### Differential ChIP-seq and overlap analysis

For JunD ChIP samples, peaks were called using the MACS2 parameters “-q 0.01 –m 5 50” selected as the most stringent values which included ChIP-qPCR validated regions. Differential binding analysis was performed with the DiffBind (ver. 2.0.9) Bioconductor package using default settings with statistical analysis of differentially-bound regions performed using the integrated edgeR analysis. Regions having FDR<0.05 were considered differential binding events. JunB co-bound regions were defined as those differentially-bound regions having at least 1 base overlap with JunB ChIP-seq peaks as reported by MACS2 (above). For occupancy comparison by venn diagram, the DiffBind “dba.plotVenn” tool was used to compare overlapping peaks from the individual Jun family member data sets for which the largest number of peaks were called by MACS2 (highest signal-to-noise).

### Microarray analysis

Publicly available microarray data was downloaded from GEO and analyzed in R using several packages available from Bioconductor. Briefly, data was read in using “simpleaffy” (ver. 2.48.0) and normalized using “gcrma” (ver. 2.44.0). Normalized data was filtered to remove control probes and low expression data using default settings of the “nsFilter” function. Differential expression analysis was performed using “limma” (ver. 3.28.21) with the “eBayes” function, default settings. Genes having an FDR<0.05 were considered differentially expressed.

### Gene set enrichment analysis

Gene sets used in gene set enrichment analysis (GSEA) were generated by taking the top 100 upregulated or downregulated genes (sorted by p-value) from edgeR DE analyses of RNA-Seq data comparing Th1 or iTreg cells with Th17 cells (for Th1 and iTreg signatures), and Th17 to Th0 (for the Th17 signature). Each gene in the *Junb* ^*+/+*^ *CD4cre* vs. *Junb* ^*fl/fl*^ *CD4cre* DE data set was assigned a rank by taking the −log10 of the DE *p*-value and multiplying by the sign of the log2 fold-change for that gene. The ranked dataset was then analyzed for enrichment of either Th1 or iTreg signatures using the java-based GSEAPre-Ranked utility, with the options “Number of permutations = 10,000”, and “Enrichment statistic = weighted”.

### Pathway analysis

 Ingenuity canonical pathway analysis (IPA, Qiagen), was performed on significantly differentially regulated genes with an FDR<0.01 based on edgeR DE analysis of RNA-Seq data comparing *Junb* ^*+/+*^ *CD4cre* vs. *Junb* ^*fl/fl*^ *CD4cre* Th17 cell differentiation cultures. This list included a total of 480 genes. Enriched pathways with a significance threshold of *p*<0.05 were reported.

### Network creation, analysis, and visualization

 Custom scripts were used to integrate RNA-Seq and ChIP-Seq outputs to compile a network of direct targets of transcription factors. Briefly, for each transcription factor-knockout RNA-Seq experiment (*Junb*, *Batf*, *Stat3*, *Irf4*, *Rorc*, and *Fosl2*), all differentially expressed genes (FDR<0.05) were added to the network and classified as “DIRECT” targets if at least one ChIP-Seq peak was found within 5 kb of the gene body, or “INDIRECT” if no proximal ChIP-Seq peaks were found. Targets were also classified as either “activation” targets (if gene expression was decreased in TF-KO), or “repression” targets (if gene expression was increased). The resulting network was visualized using Cytoscape (ver. 3.4), where various differential expression datasets comparing in vitro differentiated T helper subsets were layered into the analysis for node coloring as indicated^62^. For certain validated targets with ChIP-Seq peaks falling outside the 5 kb threshold (Ifng, Irf8, and Tbx21) “DIRECT” network edges were added manually. In addition, the resulting network table was used to analyze the net effect of transcription factor regulatory pairs on subset-preferential gene expression by comparing differences in log2 fold-change of significant targets using custom scripts in R.

### Code availability

All code is available upon request from the corresponding author.

### Data availability

Sequence data that support the findings of this study have been deposited in GEO with the primary accession code GSE98414. Other sequence data referenced in this study are listed in Supplementary Data 1. All other data that support the findings of this study are available from the corresponding author upon request.

## Electronic supplementary material

Supplementary information

Supplementary Data
