# Supplementary material for: JunB promotes Th17 cell identity and restrains alternative CD4+ T-cell programs during inflammation
Source: Nat Commun. 2017 Aug 21;8:301. doi: 10.1038/s41467-017-00380-3 (PMC5563507; doi:10.1038/s41467-017-00380-3)
Supplement: Supplementary file 1 — Supplementary information [file 41467_2017_380_MOESM1_ESM.pdf]

Title of file for HTML: Supplementary Information
Description: Supplementary Figures, Supplementary Tables

Title of file for HTML: Supplementary Data 1
Description: NGS Datasets

Supplementary Figure 1.

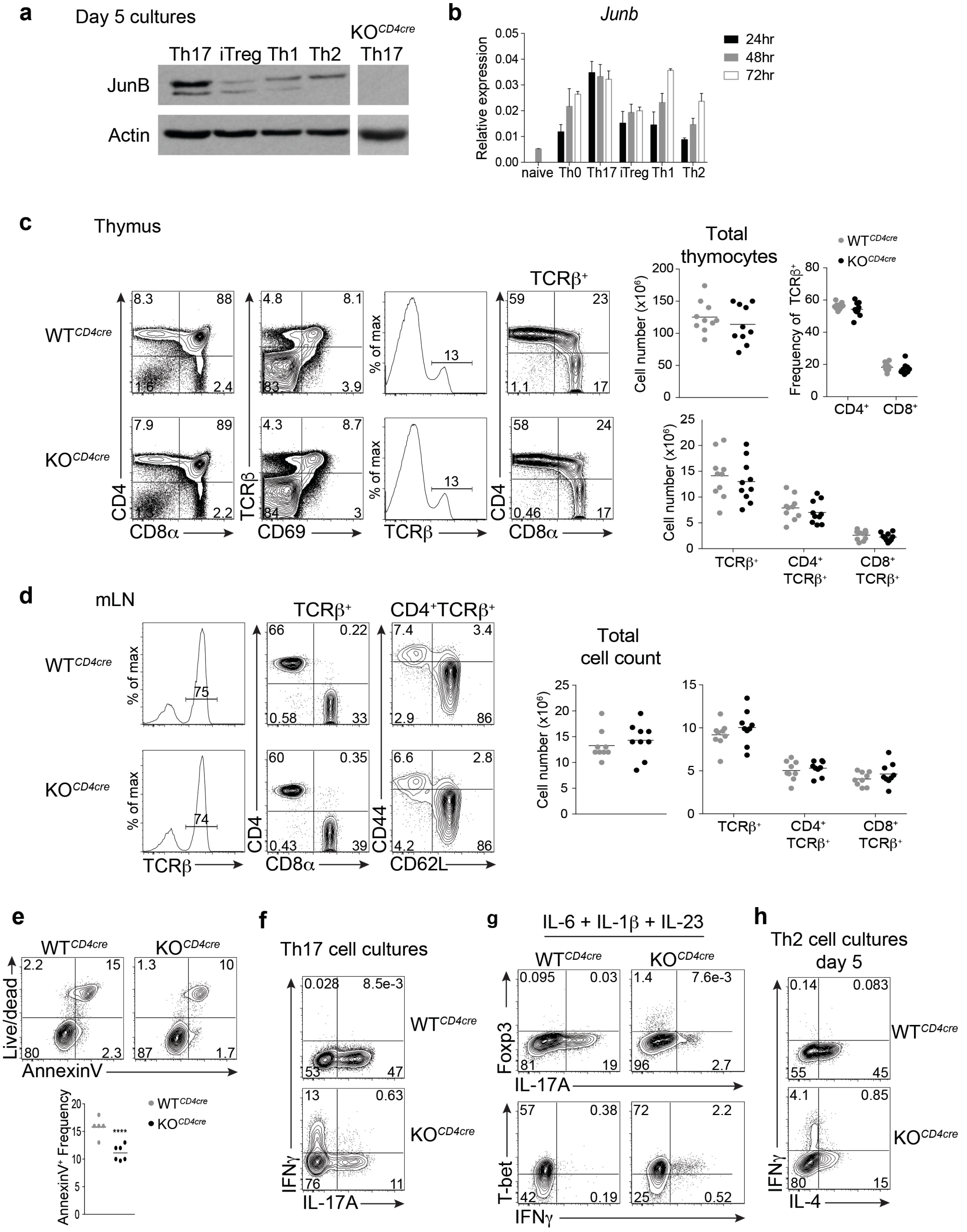

Supplementary Figure 1. JunB is dispensable for conventional $CD4^+$ αβ T cell development. (a) Western blot analysis of JunB in lysates from WT^{CD4cre} naïve $CD4^+$ T cells cultured under Th17 (75% IL-$17A^+$), iTreg (71% $Foxp3^+$), Th1 (75% $IFN\gamma^+$), or Th2 (40% IL-4^+) polarizing conditions and KO^{CD4cre} naïve $CD4^+$ T cells cultured under Th17 polarizing conditions for 5 days, with Actin as a loading control. Data are representative of two independent experiments. (b) Quantitative PCR (QPCR) analysis of the expression of *Junb* transcript in WT^{CD4cre} naïve $CD4^+$ T cells and WT^{CD4cre} Th0, Th17, iTreg, Th1, and Th2 cells at 24, 48, and 72 hours post polarization. RNA expression is presented relative to *Actb* expression. Error bars represent SEM of two independent experiments. (c) Flow cytometry of thymocytes from WT^{CD4cre} and KO^{CD4cre} mice, stained for TCRβ, CD4, CD8α, and CD69. Total thymocyte counts, frequencies of $CD4^+TCR\beta^+$ and $CD8^+TCR\beta^+$ thymocytes, and total numbers of $TCR\beta^+$ mature thymocytes are shown. (d) Flow cytometry of cells from the mesenteric lymph nodes (mLN) of WT^{CD4cre} and KO^{CD4cre} mice, stained for TCRβ, CD4, and CD8α. Total cell counts and numbers of $TCR\beta^+$ cells are shown. (e) IL-17A *versus* IFNγ expression in restimulated 72h Th17 cell cultures shown in Fig. 1c. (f) AnnexinV staining in IL-$17A^+$ cells at 72h post Th17 cell polarization. Frequency of $AnnexinV^+$ cells is displayed. (g) Flow cytometry of WT^{CD4cre} and KO^{CD4cre} naïve $CD4^+$ T cells cultured under pathogenic (IL-6,IL-1β, and IL-23) Th17 cell polarizing conditions for 72 hours followed by restimulation. Data are representative of three independent experiments. (h) Flow cytometry of WT^{CD4cre} and KO^{CD4cre} naïve $CD4^+$ T cells cultured under Th2 polarizing conditions, for 5 days followed by restimulation. Data are representative of three independent experiments.

Supplementary Figure 2.

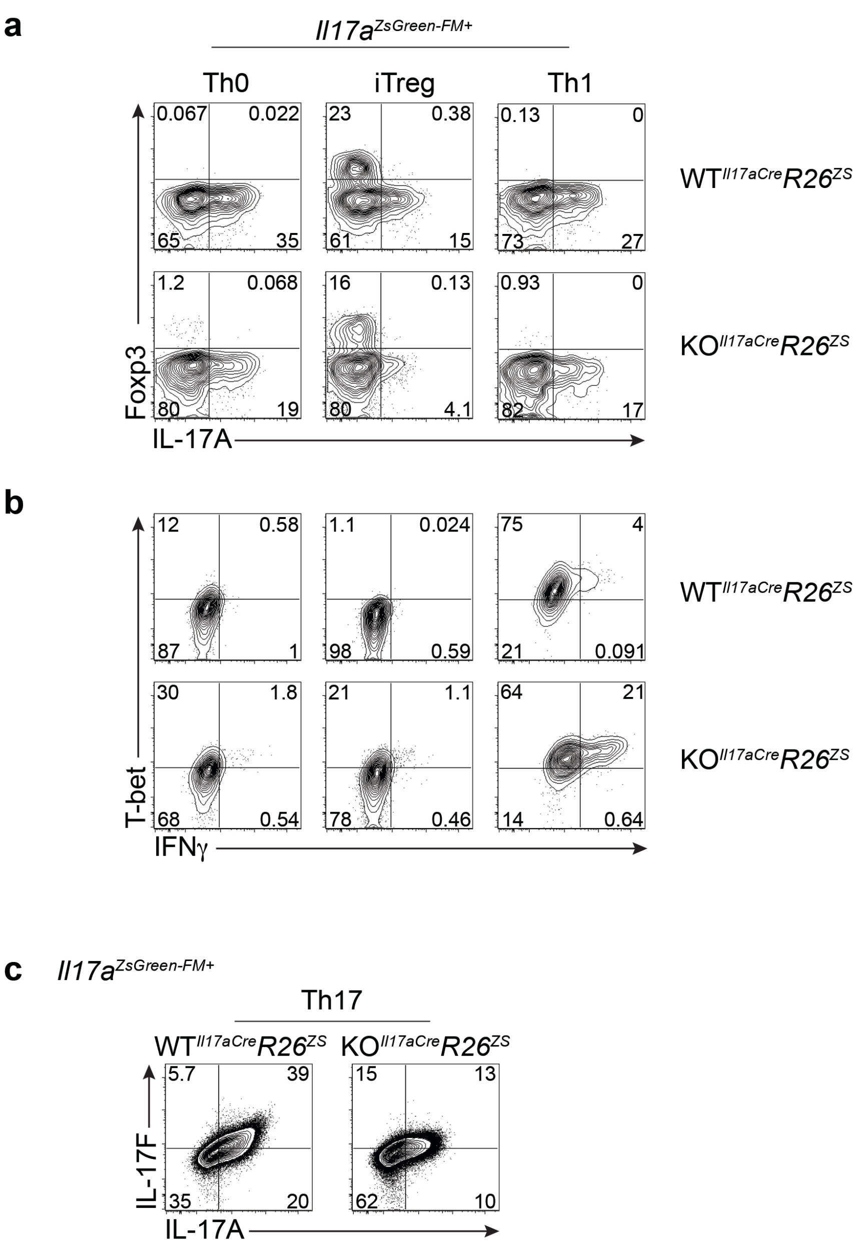

Supplementary Figure 2. JunB restricts Th17 cell plasticity. (a), (b), and (c) Flow cytometry of sort-purified *Il17a*$^{ZsGreen\text{-}FM+}$ cells from day 4 WT^{Il17aCre}*R26*^{ZS} and KO^{Il17aCre}*R26*^{ZS} Th17 cell cultures, replated in Th0, Th17, iTreg, and Th1 cell polarizing conditions for 4 days, as indicated, and followed by restimulation. Data are representative of three independent experiments.

Supplementary Figure 3.

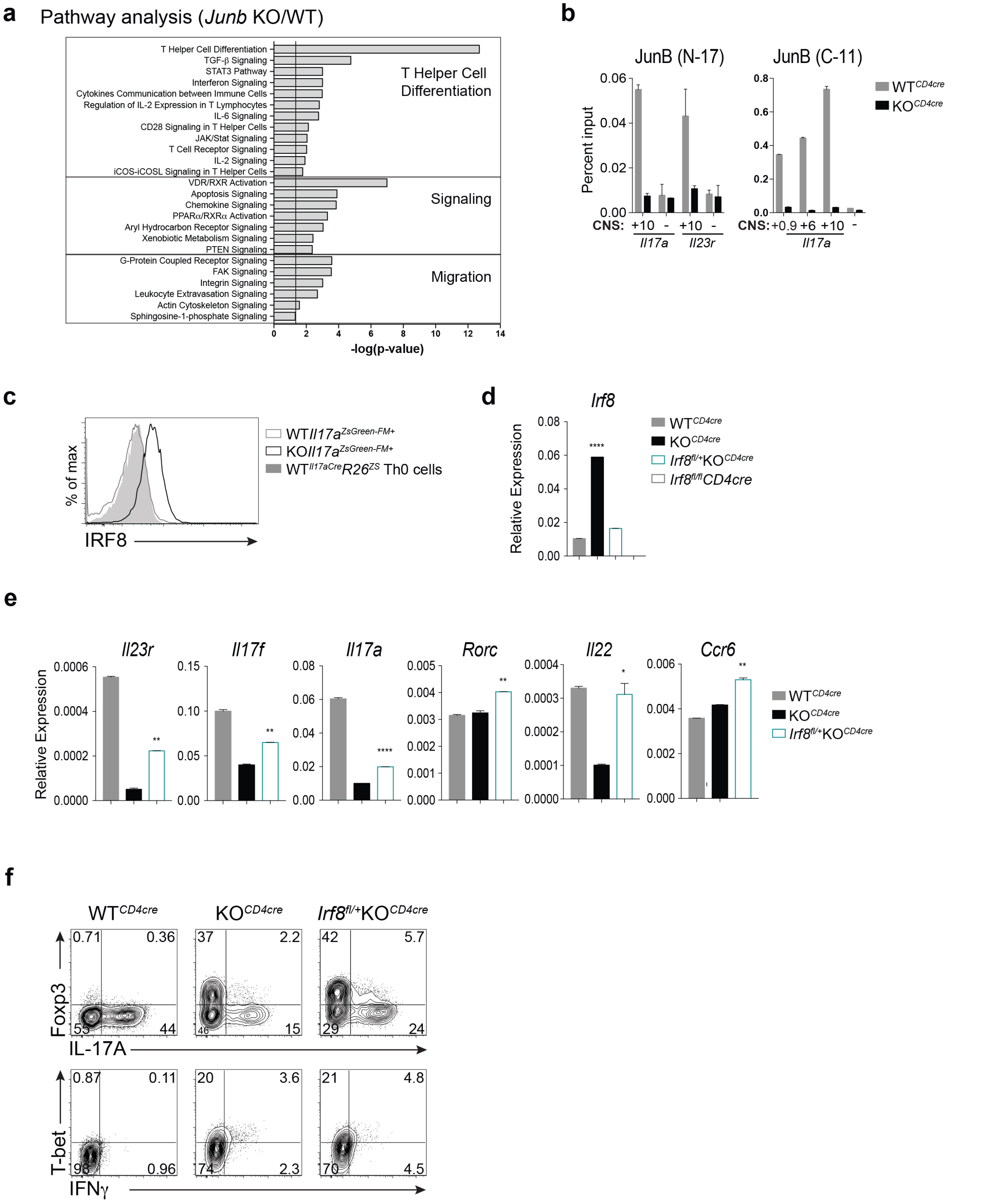

Supplementary Figure 3. Restoration of IRF8 partially rescues the KO^{CD4cre} Th17 cell program. (a) Ingenuity canonical pathway analysis for genes differentially expressed in KO^{CD4cre} relative to WT^{CD4cre} 48 hour Th17 cell polarization cultures. Line represents a significance threshold of $p<0.05$. (b) JunB ChIP antibody specificity is shown at the *Il17a* and *Il23r* loci in WT^{CD4cre} versus KO^{CD4cre} 48 hour Th17 cell polarization cultures. Data represent two independent experiments with error bars indicating SEM. (c) Flow cytometry of IRF8 expression in *WTIl17a*$^{\text{ZsGreen-FM+}}$ (grey) and *KOIl17a*$^{\text{ZsGreen-FM+}}$ (black) Th17 cells 4 days post polarization, with WT^{Il17aCre}*R26*^{ZS} Th0 cells (shaded) as a negative control. (d) QPCR analysis of *Irf8* expression in WT^{CD4cre}, KO^{CD4cre}, and *Irf8*$^{fl/+}$KO^{CD4cre} 48 hour Th17 cell polarization cultures, with *Irf8*$^{fl/fl}$*CD4cre* Th17 cells as a negative control. RNA expression is presented relative to *Actb* expression. (e) QPCR analysis of *Il23r*, *Il17f*, *Il17a*, *Rorgt*, *Il22*, and *Ccr6* expression as in Fig. 3e. (f) Flow cytometry of WT^{CD4cre}, KO^{CD4cre}, and *Irf8*$^{fl/+}$KO^{CD4cre} 72 hour Th17 cell polarization cultures. Cells were stimulated with PMA and ionomycin, and then stained for intracellular cytokines and transcription factors. Data are representative of three independent experiments, error bars indicate SEM. *$p<0.05$; **$p<0.01$; ****$p<0.0001$ (unpaired two tailed Student's *t* test)

Supplementary Figure 4.

a

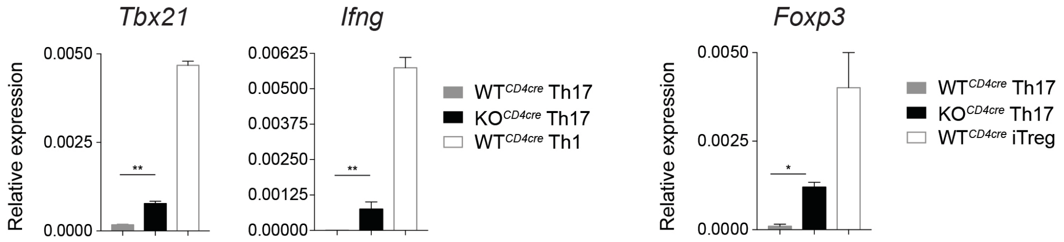

b

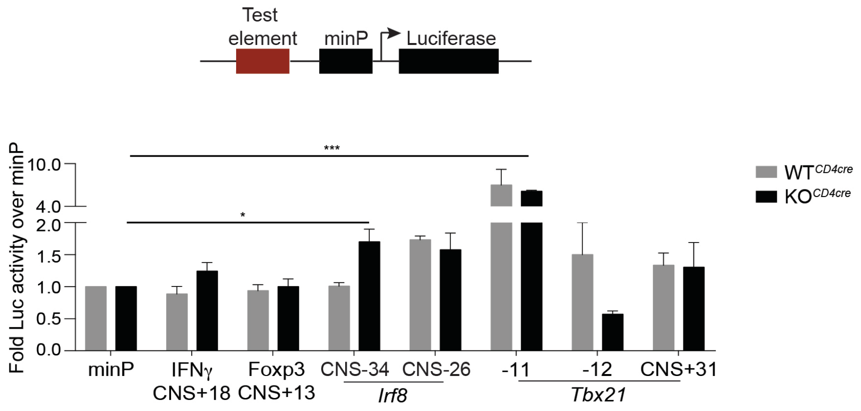

c

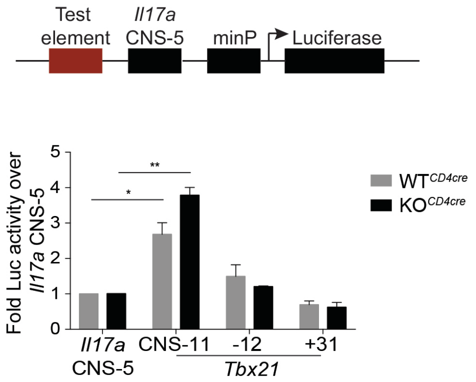

Supplementary Figure 4. Expression and function of cis elements for JunB-repressed loci. (a) QPCR analysis of *Tbx21* and *Ifng* gene expression in WT^{CD4cre} and KO^{CD4cre} 48 hour Th17 cell polarization cultures, with WT^{CD4cre} 48hour Th1 cell polarization cultures as a positive control. The right panel depicts QPCR analysis of *Foxp3* expression in WT^{CD4cre} and KO^{CD4cre} 48 hour Th17 cells, with WT^{CD4cre} 48hour iTreg cells as a positive control. RNA expression is presented relative to *Actb* expression. (b) Luciferase reporter assay of enhancer activity for select JunB-bound regions at the *Ifng*, *Foxp3*, *Irf8*, and Tbx21 loci, in WT^{CD4cre} and KO^{CD4cre} naïve $CD4^+$ T cells cultured under Th17 polarizing conditions for 48 hours. JunB-bound CNS of interest at each locus are indicated in Fig. 4a. (c) Luciferase reporter assay of silencer activity for select JunB-bound regions at the *Tbx21* locus, as in (b). All error bars represent SEM of three independent experiments. *p<0.05; **p<0.01; ***p<0.001 (unpaired two tailed Student's *t* test)

Supplementary Figure 5.

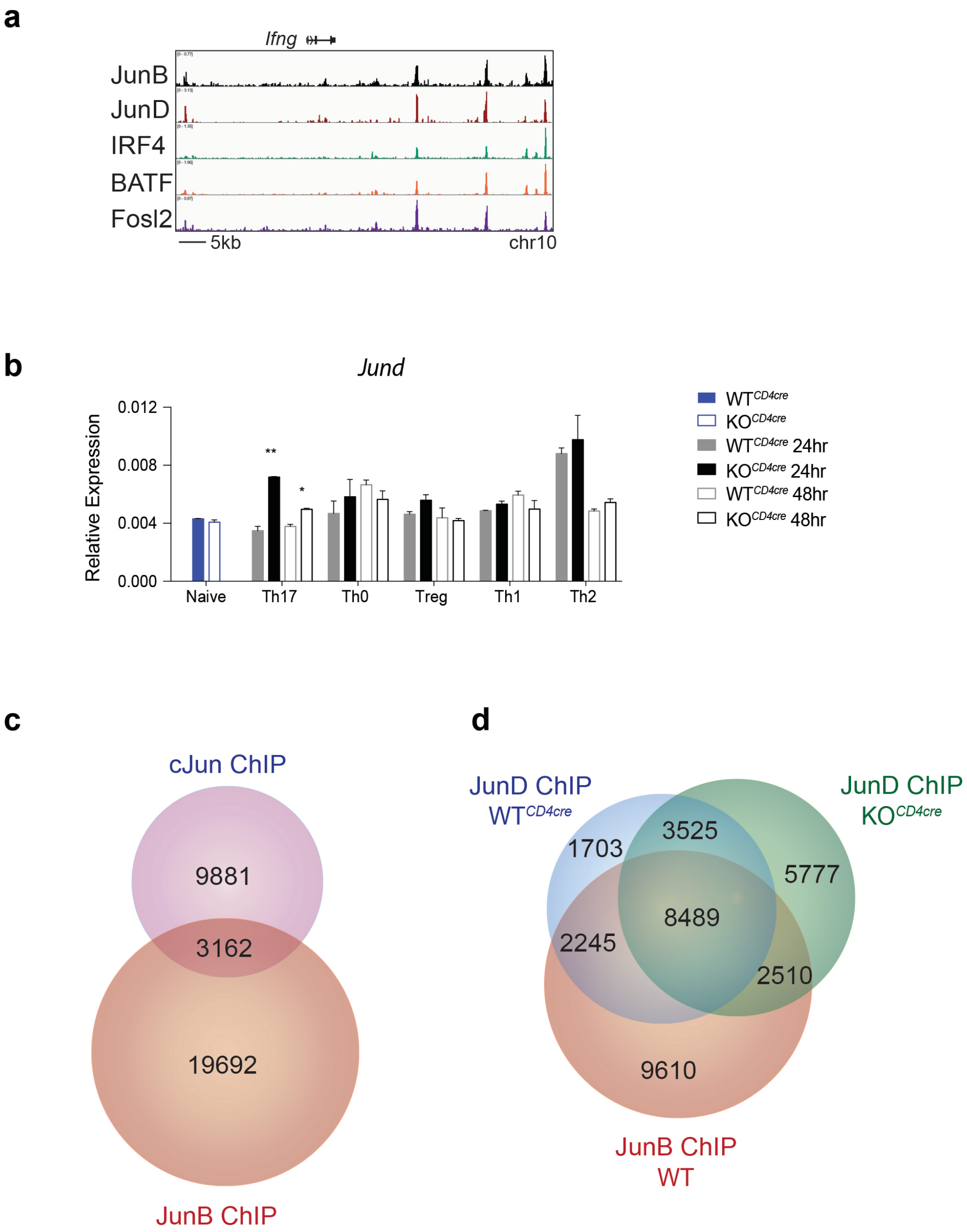

Supplementary Figure 5. Selective upregulation of *Jund* in KO^{CD4cre} Th17 cells. (a) ChIP-Seq tracks for JunB, JunD, IRF4, BATF, and Fosl2 at the *Ifng* locus in 48 hour polarized wildtype Th17 cells. (b) QPCR analysis for *Jund* transcript, relative to *Actb* expression, in $WT^{CD4\text{-}cre}$ and $KO^{CD4\text{-}cre}$ $CD4^+$ naïve T cells and $CD4^+$ naïve T cells cultured under Th17, Th0, iTreg, Th1, and Th2 cell conditions for 24 and 48 hours. Error bars represent SEM of two independent experiments. (c) Venn diagram displaying the overlap of JunB peaks with occupancy of cJun in $WT^{CD4\text{-}cre}$ Th17 cell polarization cultures. Peaks considered in this analysis were called significant by MACS2 with q-value < 0.00005. (d) Venn diagram displaying the overlap of JunB peaks with occupancy of JunD in 48h $WT^{CD4\text{-}cre}$ and $KO^{CD4\text{-}cre}$ Th17 cell polarization cultures. Peaks considered in this analysis were called significant by MACS2 with q-value < 0.01. *p<0.05; **p<0.01 (unpaired two tailed Student's *t* test)

Supplementary Figure 6.

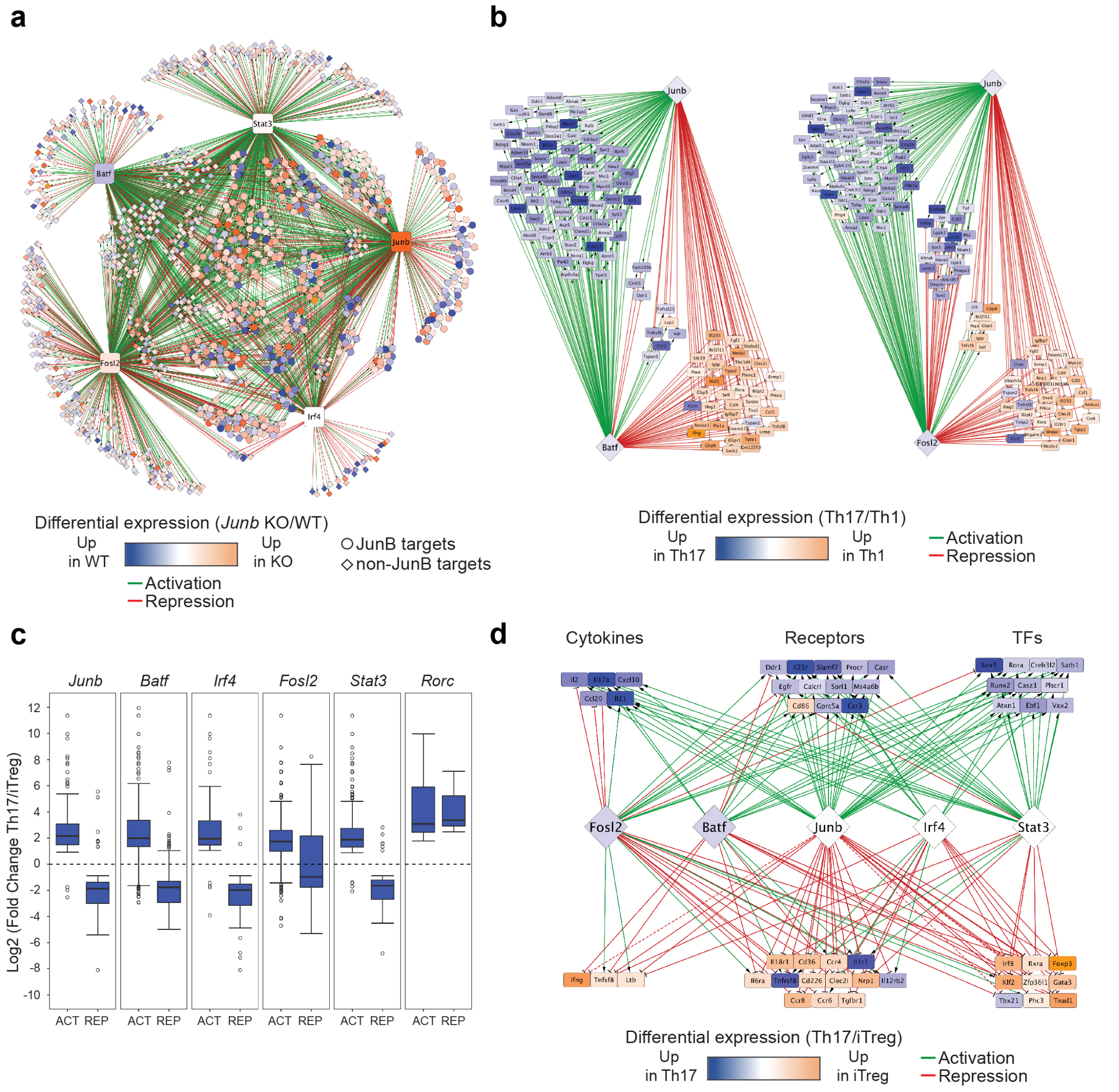

Supplementary Figure 6. Analysis of shared targets of JunB, BATF, and Fosl2. (a) Global network view showing direct targets of JunB, BATF, Fosl2, IRF4, and STAT3. Nodes are colored based on differential expression (DE) in *Junb* WT *versus* KO. (b) Network representation of shared targets of JunB and BATF or Fosl2. Nodes are colored based on log2FC of DE in Th17 *versus* Th1, only DE genes that are direct targets are shown. (c) Comparison of the Th17 *versus* iTreg preference of activation and repression targets of Th17 TFs. For each category, the log2FC for genes with DE in Th17 *versus* iTreg (FDR < 0.05) is plotted. (d) Network representation of Th17 TF targets with functional categorization. Node coloring indicates log2FC of DE in Th17 *versus* iTreg (FDR < 0.05). Genes were classified and grouped based on molecular function. Only targets with 2 or more edges are shown. Dashed lines indicated direct targets validated using ChIP-QPCR and luciferase assay that fall outside the 5 kb threshold for global target classification.

Supplementary Figure 7.

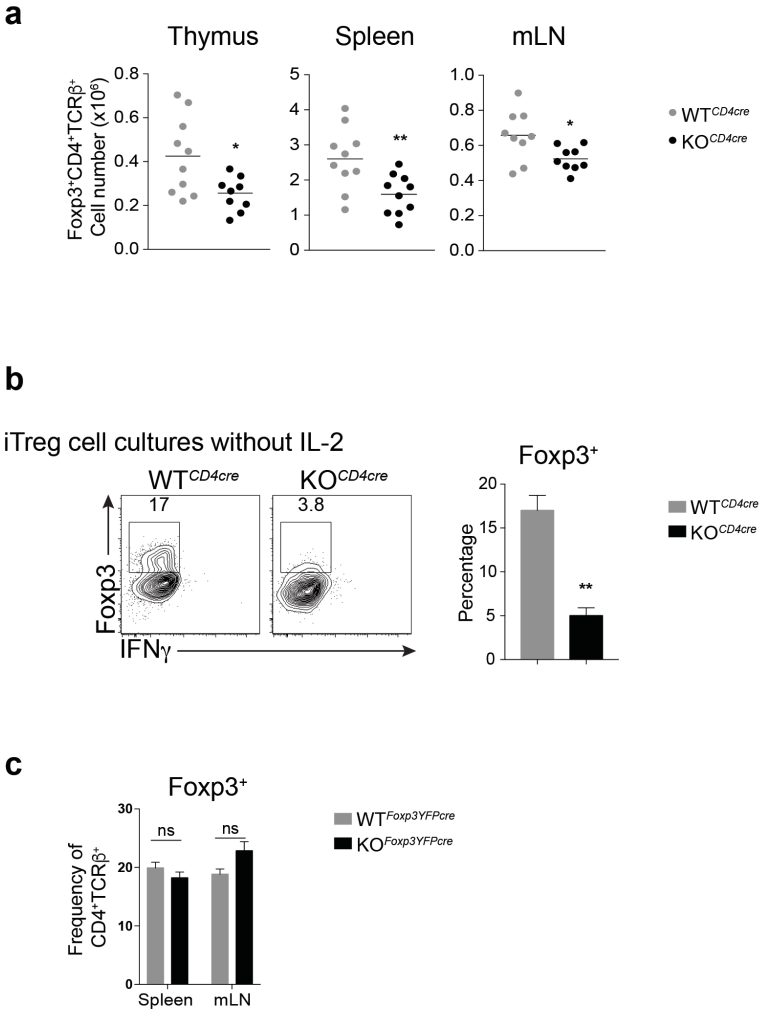

Supplementary Figure 7. Foxp3$^+$CD4$^+$TCRβ$^+$ cells are reduced in the absence of JunB. (a) Total numbers of Foxp3$^+$CD4$^+$TCRβ$^+$ cells from WT^{CD4cre} and KO^{CD4cre} thymus, spleen, and mLN are displayed. (b) Foxp3 expression in 48h iTreg polarization cultures with TGFβ alone and no exogenous IL-2. Error bars represent SEM of two independent experiments with at least 5 mice per group. (c) Frequency of Foxp3$^+$ CD4$^+$ TCRβ$^+$ Treg cells in the spleen and mesenteric LN from *Junb*$^{+/+}$ *Foxp3*$^{\text{YFP-Cre}}$ and *Junb*$^{\text{fl/fl}}$ *Foxp3*$^{\text{YFP-Cre}}$ mice. Error bars represent SEM of three independent experiments with at least 11 mice per group. *p<0.05; **p<0.01 (unpaired two tailed Student's *t* test)

Supplementary Figure 8.

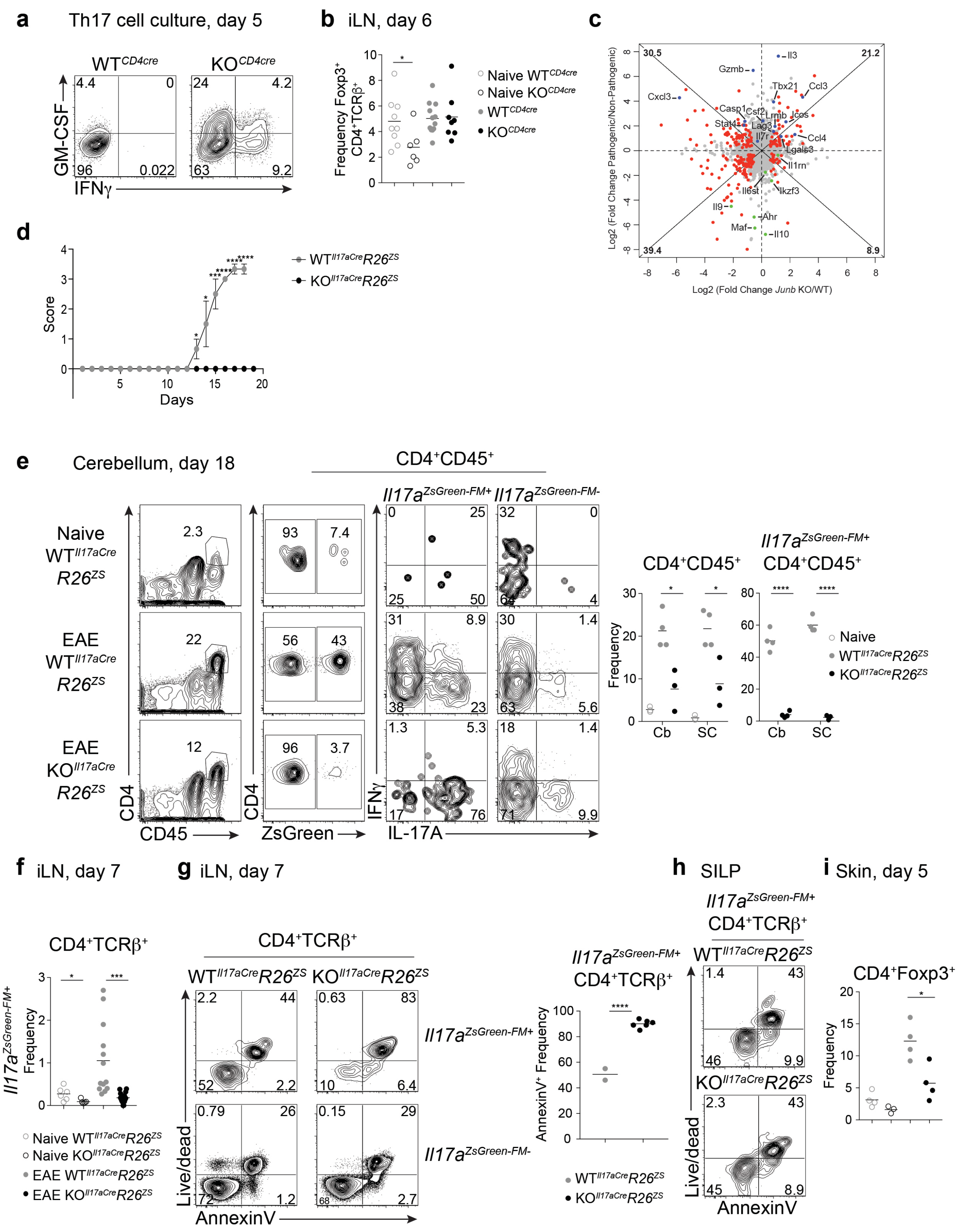

Supplementary Figure 8. JunB is required for the maintenance of inflammatory Th17 cells in vivo. (a) Flow cytometry of IFNγ and GM-CSF production in 5 day Th17 cell polarization cultures of WT^{CD4cre} and KO^{CD4cre} $CD4^+$ T cells, restimulated prior to staining. (b) Frequency of $Foxp3^+CD4^+TCR\beta^+$ cells in the draining lymph node of WT^{CD4cre} and KO^{CD4cre} naïve mice at day 6 post-induction of EAE. (c) JunB does not globally regulate a pathogenic Th17 cell signature. Comparison of differential expression (DE) targets of JunB with those of Th17 cell cultures executed under pathogenic (IL-6, IL-23, and IL-1β) versus non-pathogenic (IL-6 and TGF-β) conditions. Genes highlighted in red show DE (FDR < 0.05) in both datasets. Numbers in corners indicate the percentage of all significant genes that fall within that quadrant. Key effector genes representative of pathogenic and non-pathogenic signatures are highlighted in blue and green, respectively. (d) EAE disease scores for $WT^{Il17aCre}R26^{ZS}$ and $KO^{Il17aCre}R26^{ZS}$ mice, error bars are SEM of 5 mice per cohort. (e) Flow cytometry of IL-17A, IFNγ, and Foxp3 expression in *ex vivo* restimulated $Il17a^{ZsGreen\text{-}FM+}CD4^+CD45^+$ cells isolated from the cerebellum of $WT^{Il17aCre}R26^{ZS}$ and $KO^{Il17aCre}R26^{ZS}$ mice, when $WT^{Il17aCre}R26^{ZS}$ mice were at peak disease. Frequencies of total $CD4^+CD45^+$ cells in the cerebellum and spinal column of $WT^{Il17aCre}R26^{ZS}$ and $KO^{Il17aCre}R26^{ZS}$ mice, as well as naïve control, when $WT^{Il17aCre}R26^{ZS}$ mice were at peak disease are shown. (f) Frequencies of $Il17a^{ZsGreen\text{-}FM+}CD4^+TCR\beta^+$ cells in the draining lymph node of $WT^{Il17aCre}R26^{ZS}$ and $KO^{Il17aCre}R26^{ZS}$ mice day 6 post induction of EAE, with naive $WT^{Il17aCre}R26^{ZS}$ and $KO^{Il17aCre}R26^{ZS}$ mice as controls. (g) Flow cytometry of AnnexinV staining in $Il17a^{ZsGreen\text{-}FM+}CD4^+TCR\beta^+$ cells from the draining lymph node of $WT^{Il17aCre}R26^{ZS}$ and $KO^{Il17aCre}R26^{ZS}$ mice day 6 post induction of EAE. Frequencies of $AnnexinV^+$ cells among $Il17a^{ZsGreen\text{-}FM+}CD4^+TCR\beta^+$ cells are shown. (h) Flow cytometry *of AnnexinV staining in* $Il17a^{ZsGreen\text{-}FM+}CD4^+TCR\beta^+$ *cells from the SILP of naïve* $WT^{Il17aCre}R26^{ZS}$ and $KO^{Il17aCre}R26^{ZS}$ mice. (i) Frequencies of $Foxp3^+$ *ex vivo* restimulated cells harvested from the skin day 5 post infection. *p<0.05; ***p<0.001; ****p<0.0001 (unpaired two tailed Student's *t* test)

Supplementary Figure 9.

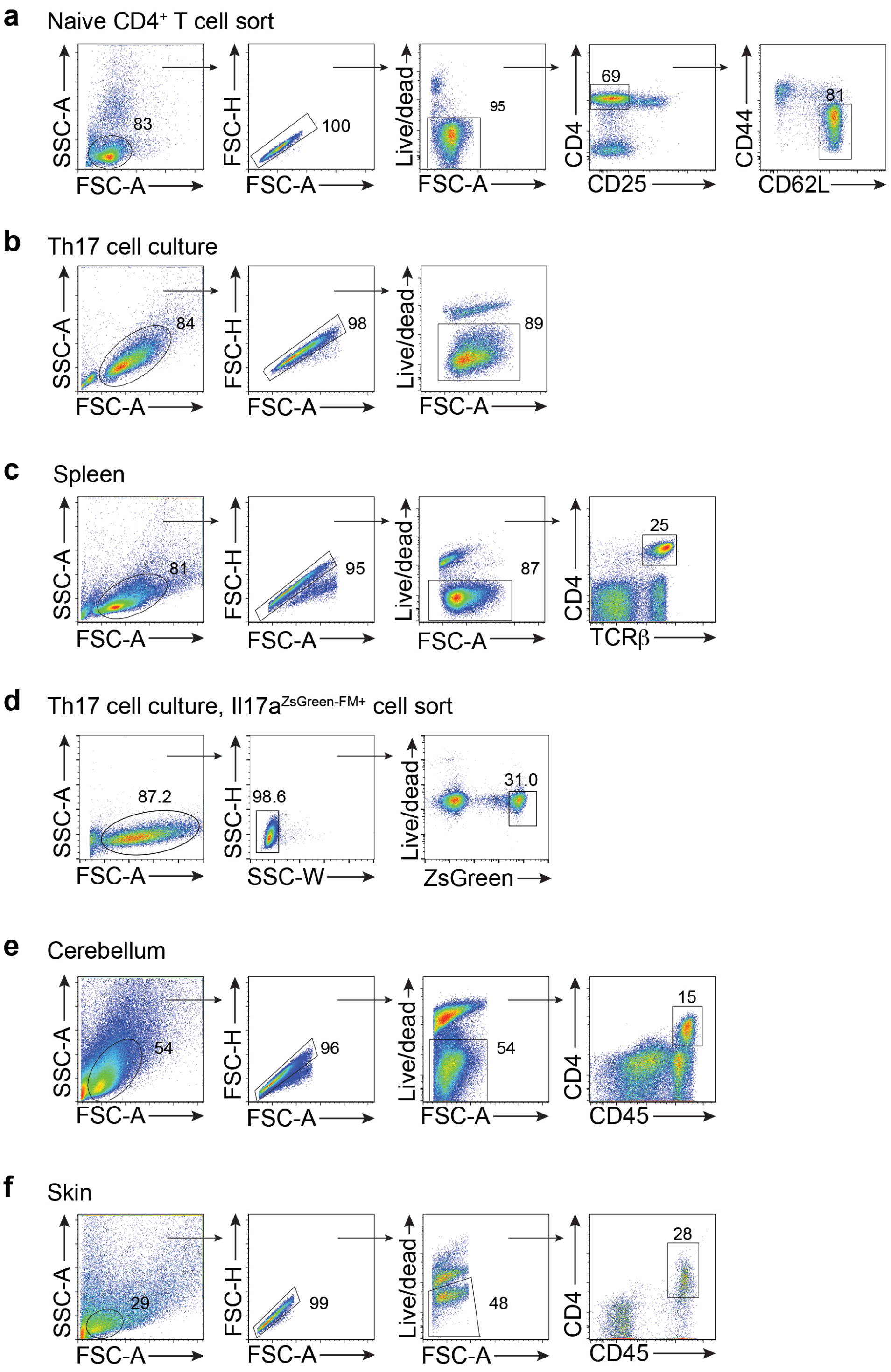

Supplementary Figure 9. Flow cytometry sorting and gating strategies. (a) Naïve CD4+ T cell sort, used for all *in vitro* cell cultures (related to all figures except Fig. 8 and Supplementary Fig. 8). (b) Gating strategy for flow cytometry analysis of all *in vitro* cell cultures (Fig. 1, 2, and 3 and Supplementary Fig. 1, 2, 3, 7, and 8). (c) Flow cytometry gating scheme for peripheral analysis of steady state mice (Fig. 1 and 7 and Supplementary Fig. 1 and 7. (d) Sorting gates used for $\textit{Il17a}^{ZsGreen\text{-}FM+}$ *in vitro* cell cultures (Fig. 2 and Supplementary Fig. 2). (e) Flow cytometry gating scheme for cells harvested from the cerebellum and (f) skin (Fig. 8 and Supplementary Fig. 8).

Supplementary Figure 10.

a JunB

WT^{CD4cre} KO^{CD4cre}

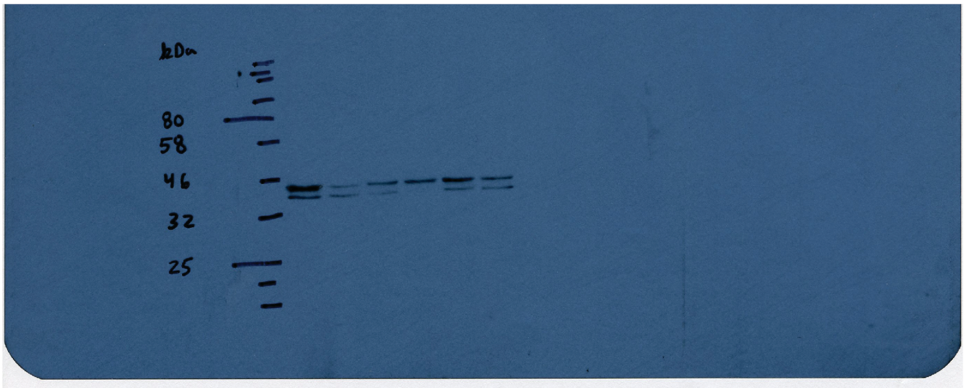

b Actin

WT^{CD4cre} KO^{CD4cre}

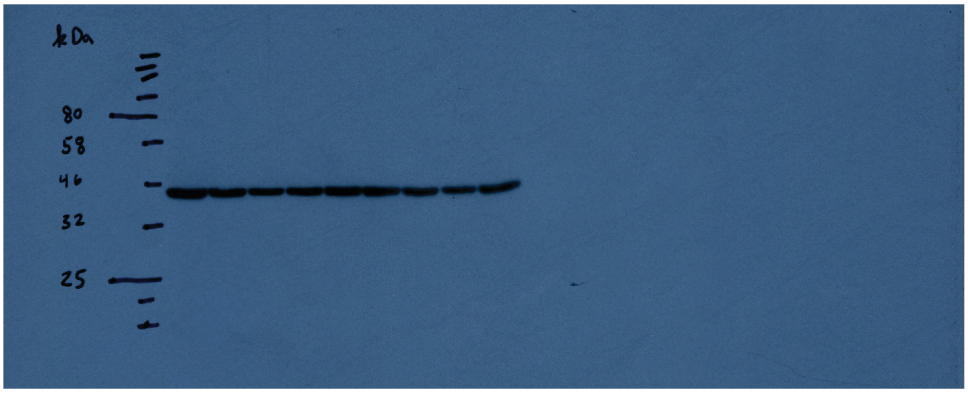

Supplementary Figure 10. JunB expression in CD4$^+$ Th cell subsets. Western blot analysis of (a) JunB and (b) Actin in lysates from CD4$^+$ Th cell *in vitro* cultures, at day 5 post polarization (related to Supplementary Fig. 1)

Supplementary Table 1. AP-1 consensus sequences located within select JunB-bound regions

Locus	AP-1 consensus sequence
Il17a CNS+0.9	TGACTCA
Il17a CNS+6	TGAGTCA
Il17a CNS+10	TGAGTCA….TTAGTCA
Irf8 CNS-34	TGACTCA
Irf8 CNS-26	TGAGTCA
Tbx21 CNS-11	TGAGTCA
Tbx21 CNS-12	TGAGT….TGAGA
Tbx21 CNS+31	TGAGTCA
Ifng CNS+18	TGACTCA….TGAGTCA
Foxp3 CNS+13	TGACTCA
Il23r CNS+8	TGAGTCA

Supplementary Table 2. QPCR primers

Transcript	Forward primer	Reverse primer
Il23r	CAAGAGACACTGATTTGTGGGAAAGACA	AGTGTTCCAGGTGCATGTCATGTT
Il17f	AGAAGCAGCCATTGGAGAAACCAG	CCTCAGAATGGCAAGTCCCAACAT
Il17a	AGAAGGCCCTCAGACTACCTCAA	AGCTTCCCAGATCACAGAGGGATA
Rorγt	ACAAGTCATCTGGGATCCACTACG	AGGAGTAGGCCACATTACACTGCT
Il22	TACATGCAGGAGGTGGTGCCTTTC	CATTCTTCTGGATGTTCTGGTCGTCACC
Ccr6	TTTAACTGTGGGATGCTGCTCCTG	GAGCGTACCCGGAAAGATTTGGTT
Tbet	AGCTCACCAACAACAAGGGGGCTT	TCCACGATGTGCAGCCGGGG
Ifnγ	ACCTTCTTCAGCAACAGCAAGGCG	TGGCGCTGGACCTGTGGGTT
Foxp3	GCTGGAAGACTGCACCCAAGGGC	GCCCCACTTCGCAGGTCCCG
Junb	GGAGCGCATCAAAGTGGAGCGAAA	AGCCTTGAGTGTCTTCACCTTGTCCT
Fosl2	TGAACTCTCCAGGCAGGACCTTAT	ATGCTTCCGAGCAGAGATACTCCT
Irf4	CAAGCAGGACTACAATCGTGAGGA	AGTAGGAGGATCTGGCTTGTCGAT
Batf	AGACACAGAAAGCCGACACCCTTCA	ACTTGAGCTCCTCGGTGAGCTGTTT
cJun	GGCTGCAAGCCCTGAAGGAAGAG	TTGATCCGCTCCTGAGACTCCATGT
Jund	ACGCAGTTCCTCTACCCGAAGGTG	TGGCTTTGCTTGTGCAGGTCCTC

Supplementary Table 3. Primers used to clone select JunB-bound regions for evaluation in luciferase reporter assay

Locus	Forward primer	Reverse primer
Il17a CNS+0.9	CTATCTGGAGGATAGCTGGTGTA	TGTGACTGAGGACAATCATGTG
Il17a CNS+6	AGAGATCAGGATTCCCTCTCAG	GCTTCCTCTTCCCAAAGAAATTA
Il17a CNS+10	CACACTCTCAGTACAAGTTCTTATC	TTGATGGCAGCACATTCATATC
Irf8 CNS-34	GGGTCTGCCTTCTTGTTCTG	GTTCACACCTAAAGCACTCCA
Irf8 CNS-26	TCTCTTGCTGTCAGGTCAGAGG	GCACTCGCTTTATTAGTGGCTTCTC
Tbx21 CNS-11	TTTCCCAGCTTCGAGGAAAC	ACGGACTACAGGTTCTTCCT
Tbx21 CNS-12	GACTCTGTCTGTCTTGCTAGG	GGCATGAAAGTCTGGAGTATCT
Tbx21 CNS+31	TCTTCCAGAGTTCCCTCTCAA	CTCCCAATCCCGTCATGTTC
Ifng CNS+18	GTCTACATGAGATCAAGACCATAA	GGATAGACAAGTCACCAAGTC
Foxp3 CNS+13	GCAGAAAGATGACAGACACCATC	GCGTATGATCAGTTATGCCTGTG

Supplementary Table 4. ChIP-QPCR primers

Locus	Forward primer	Reverse primer
Il17a CNS+0.9	ACATGTTTGACTGTGCACGAGGTTTAC	CTCAGCCTGTTAGCTCATGAGTCAGT
Il17a CNS+6	TCAGAAGGGAAGCGGCAAGAAAGA	AAGCTTGAAACTGACCTGTTGGCAAAT
Il17a CNS+10	TCCCTCACATACCTCATGCTGAATTACC	GGCAGACATATGGGCATGAGCAAAG
Il17a Neg. ctrl.	TCCGTCCCAGGAAGTGTTCTCAGATA	GACAACAAAGGGACAGATGACCAAGGA
Irf8 CNS-34	CAGGTGTGTCCTCTAGGCTTTGT	ACTTCCTACCAATGCCACCTGT
Irf8 CNS-26	CAGCCGTCTCACCTCTCACAAG	CGATGACATTTCCCTCAGTCTGTCTC
Irf8 Neg. ctrl.	AGCCGGCTGAGAAAGCCACTTC	GCTGAAAGCCCAGCAGGACAGAA
Tbx21 CNS-11	AGGAGTTCTGTGGTTCTGGCTTTG	CAGCTTCGAGGAAACACCTCCTTT
Tbx21 CNS-12	GGAGTATCTCACTCCTAAGGGAAGAAGC	CGGTCTCAGTTTCCCATTCTCACTCTAC
Tbx21 Neg. ctrl.	AGCCAAGGTACACACAACCCAAGA	AGCTGCCTCAGACAAAGACACAAGTAAG
Ifng CNS+18	GGGTACCTGAGGTGAATCAGGAAAG	CGAGGAGCTGTGGTTTGTAAGTGA
Ifng Neg. ctrl.	TGGATTCCAGGCAAGACCAATACCC	GGTGACTAATGTTCCCTTCTCCCAGTTT
Foxp3 CNS+13	GCCTGCCTTGGTACATTCGTGAACT	ATAGTGCCCGTGGTTCCAGATGTT
Foxp3 Neg. ctrl.	GTCTCTGCATGTCTGTCTGTCTGT	GAGACAGTGAGAGCAGTTTAGAGGAAA
Il23r CNS+8	ATATCCTGTATCAAAGCGGCCCAG	CTGGGTTCTTGGCAAACTTCCTTC
Il23r Neg. ctrl.	TCCAGATTGCCTGACTGTATACCC	GAAGATGCACTTCTAGAAACCCGC
